# Supplementary material for: Development of SYK NanoBRET cellular target engagement assays for gain–of–function variants
Source: Front Chem Biol. Author manuscript; Available in PMC 2026 Apr 18. (PMC13089524; doi:10.3389/fchbi.2024.1447622)
Supplement: SI file [file NIHMS2163057-supplement-SI_file.pdf]

Supplementary Material

Development of SYK NanoBRET Cellular Target Engagement Assays for Gain–of–Function Variants

Jacob L. Capener[1], James D. Vasta[2], Vittorio L. Katis[3], Ani Michaud[2], Michael T. Beck[2], Sabrina C. D. Daglish[1], Sarit Cohen-Kedar[4,5], Efrat Shaham Barda[4,5], Stefanie Howell[1], Iris Dotan[4,5], Matthew B. Robers[2], Alison D. Axtman[1], Frances M. Bashore[1*]

[1] Structural Genomics Consortium, Division of Chemical Biology and Medicinal Chemistry, Eshelman School of Pharmacy, The University of North Carolina at Chapel Hill, Chapel Hill, NC 27599, USA.

[2] Promega Corporation, 2800 Woods Hollow Road, Madison, WI 53711, USA.

[3] ARUK Oxford Drug Discovery Institute, Centre for Medicines Discovery, Nuffield Department of Medicine, University of Oxford, Oxford OX3 7FZ, UK.

[4] Division of Gastroenterology, Rabin Medical Center, Petah-Tikva, Israel.

[5] Felsenstein Medical Research Center, Faculty of Medical & Health Sciences, Tel-Aviv University, Tel-Aviv, Israel.

*** Correspondence:**
Corresponding Author: frances.bashore@unc.edu

Table of Contents

1 Supplementary Figures and Tables

1.1 Supplementary Figures **S2–S10**

1.2 Chemistry Experimental **S10**

1.3 General Information for Chemical Synthesis **S10**

1.4 Abbreviations **S10**

1.5 Chemistry Schemes **S11–S12**

1.6 Chemistry Experimental Data **S13–S21**

1.7 Purity Traces and Spectra **S22–S40**

1 Supplementary Figures and Tables

1.1 Supplementary Figures

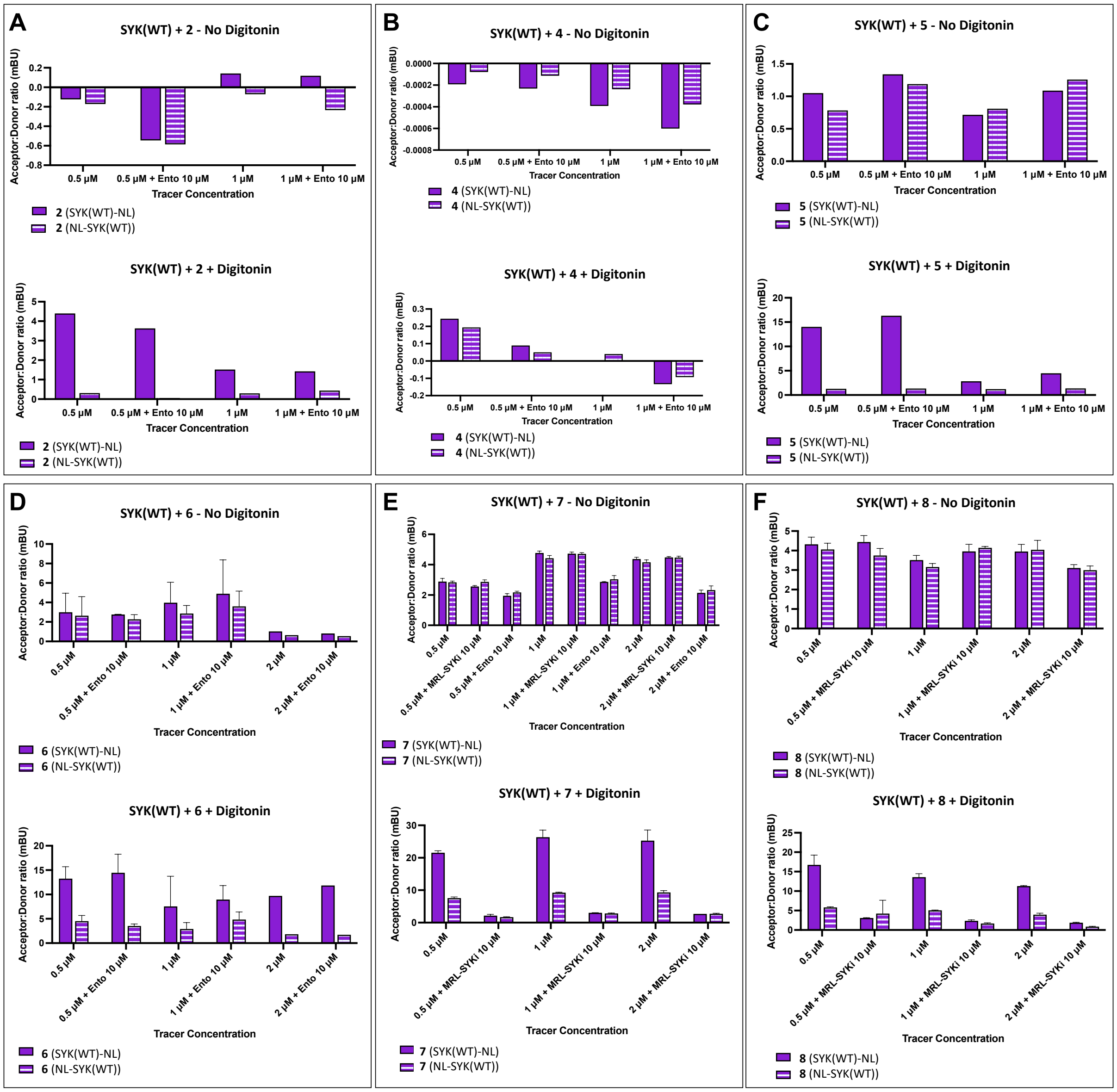

Supplementary Figure 1. SYK NanoBRET tracers **2**, **5**, **6**, **7**, and **8** generate a BRET signal (measured in mBU) with SYK(WT)-NL upon digitonin permeabilization (**A**, **C**–**F**), but not without digitonin. (**B**) Tracer **4** does not generate a BRET signal ± digitonin. Data reported are from a single experiment (**A**–**C**). Data are reported in biological duplicate ± standard deviation (**D**). Data are reported with 1–2 biological replicates ± standard deviation (**E**, **F**).

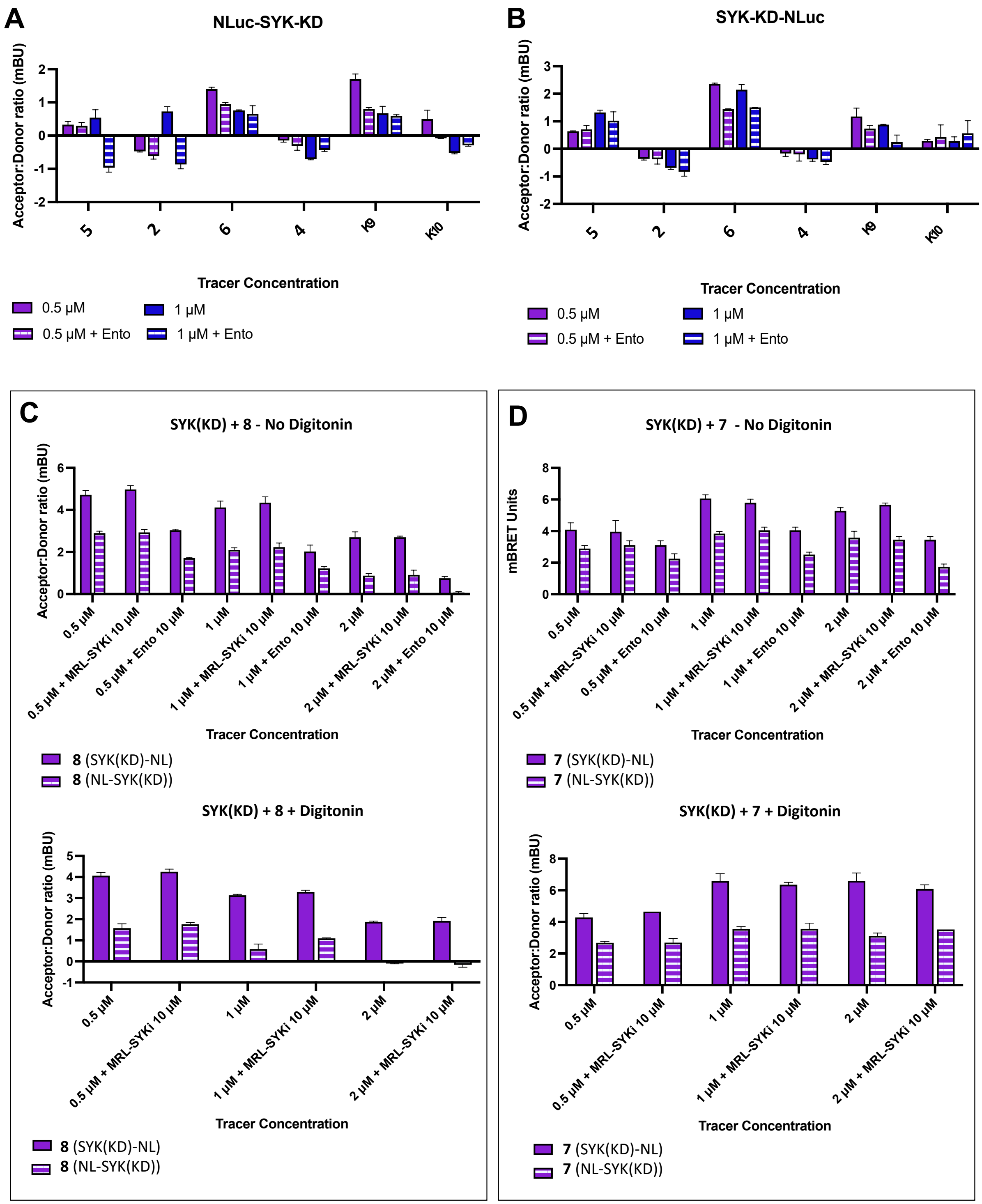

Supplementary Figure 2. SYK NanoBRET tracers **2**, **4**, **5**, **6**, **7**, **8, K9**, and **K10** did not generate a BRET signal (mBU) with SYK(KD)-NL or NL-SYK(KD) ± digitonin (**A**–**D**). Data are reported as technical duplicates ± standard deviation (**A**–**D**).

A

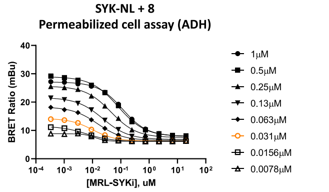

1μM	0.5μM	0.25μM	0.13μM	0.063μM	0.031μM	0.0156μM	0.0078μM
0.1160	0.08067	0.04454	0.02297	0.01500	0.01014	0.006042	0.01218

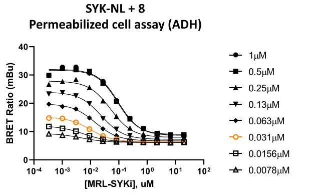

1μM	0.5μM	0.25μM	0.13μM	0.063μM	0.031μM	0.0156μM	0.0078μM
0.09703	0.09084	0.05142	0.02412	0.01449	0.009823	0.005907	0.004837

B

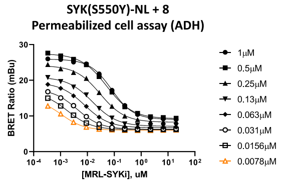

1μM	0.5μM	0.25μM	0.13μM	0.063μM	0.031μM	0.0156μM	0.0078μM
0.07911	0.05251	0.02999	0.01529	0.007911	0.004136	0.001850	0.001100

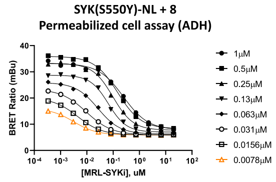

1μM	0.5μM	0.25μM	0.13μM	0.063μM	0.031μM	0.0156μM	0.0078μM
0.2462	0.1495	0.09825	0.06566	0.02624	0.009585	0.004440	0.003152

C

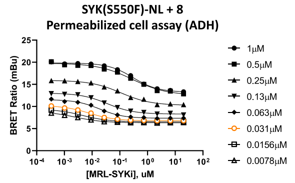

1μM	0.5μM	0.25μM	0.13μM	0.063μM	0.031μM	0.0156μM	0.0078μM
0.2991	0.2809	0.1102	0.03496	0.01849	0.007820	0.004570	0.002387

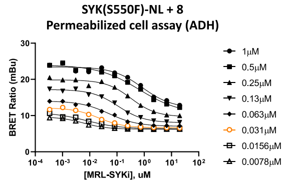

1μM	0.5μM	0.25μM	0.13μM	0.063μM	0.031μM	0.0156μM	0.0078μM
0.8527	0.6568	0.3262	0.1093	0.05096	0.02068	0.007451	0.005868

D

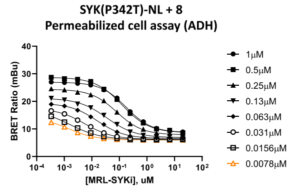

1μM	0.5μM	0.25μM	0.13μM	0.063μM	0.031μM	0.0156μM	0.0078μM
0.1783	0.1261	0.08052	0.03527	0.01596	0.005601	0.001563	0.001436

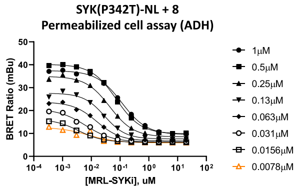

1μM	0.5μM	0.25μM	0.13μM	0.063μM	0.031μM	0.0156μM	0.0078μM
0.1149	0.07369	0.04362	0.02902	0.01546	0.006368	0.003587	0.007826

E

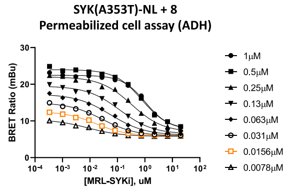

1μM	0.5μM	0.25μM	0.13μM	0.063μM	0.031μM	0.0156μM	0.0078μM
1.059	0.7680	0.3282	0.1236	0.05597	0.02784	0.01689	0.008468

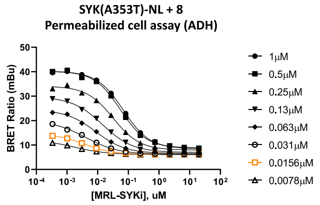

1μM	0.5μM	0.25μM	0.13μM	0.063μM	0.031μM	0.0156μM	0.0078μM
0.06818	0.05489	0.03206	0.01450	0.008647	0.004024	0.003702	0.001751

F

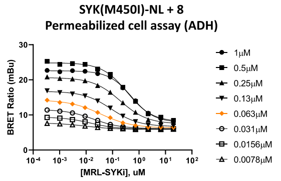

1μM	0.5μM	0.25μM	0.13μM	0.063μM	0.031μM	0.0156μM	0.0078μM
0.5212	0.3575	0.1953	0.08893	0.03536	0.01871	0.01463	0.01022

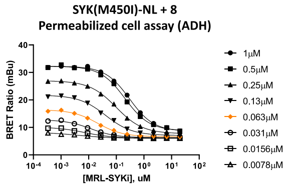

1μM	0.5μM	0.25μM	0.13μM	0.063μM	0.031μM	0.0156μM	0.0078μM
0.3014	0.2071	0.09867	0.04076	0.02122	0.01191	0.007194	0.004140

Supplementary Figure 3. SYK NanoBRET assay with titrations of tracer **8** in cells permeabilized with digitonin. An 11-point dose–response study of MRL-SYKi from a top concentration of 20 μM was used to compete away the BRET signal. Two biological replicates (left and right) and the results plotted using a log(inhibitor) vs. response (three parameter) fit. IC_{50} data are reported in μM. (**A**)

SYK(WT)-NL (**B**) SYK(S550Y)-NL (**C**) SYK(S550F)-NL (**D**) SYK(P342T)-NL (**E**) SYK(A353T)-NL (**F**) SYK(M450I)-NL.

A

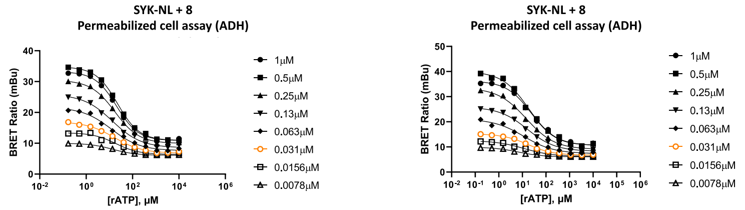

1μM	0.5μM	0.25μM	0.13μM	0.063μM	0.031μM	0.0156μM	0.0078μM
20.52	22.55	17.39	13.52	12.08	16.26	19.15	13.95

1μM	0.5μM	0.25μM	0.13μM	0.063μM	0.031μM	0.0156μM	0.0078μM
23.96	18.45	14.52	16.09	11.41	17.31	11.77	6.581

B

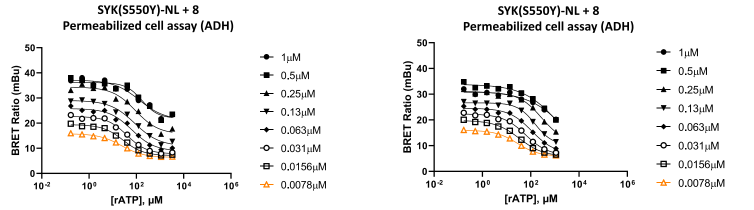

1μM	0.5μM	0.25μM	0.13μM	0.063μM	0.031μM	0.0156μM	0.0078μM
135.2	173.9	92.01	85.63	64.83	39.78	27.60	23.28

1μM	0.5μM	0.25μM	0.13μM	0.063μM	0.031μM	0.0156μM	0.0078μM
14302	1686	280.6	152.2	111.2	61.27	34.14	27.95

C

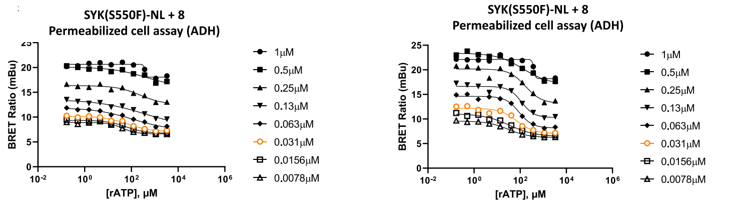

1μM	0.5μM	0.25μM	0.13μM	0.063μM	0.031μM	0.0156μM	0.0078μM
382.9	427.9	238.3	386.2	111.2	68.52	59.77	42.12

1μM	0.5μM	0.25μM	0.13μM	0.063μM	0.031μM	0.0156μM	0.0078μM
389.1	135.5	168.6	114.1	89.22	71.03	33.83	30.06

D

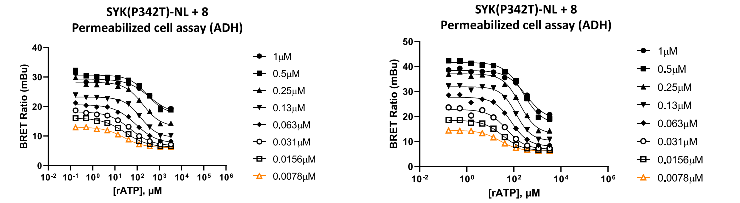

1μM	0.5μM	0.25μM	0.13μM	0.063μM	0.031μM	0.0156μM	0.0078μM
352.1	282.0	180.2	143.1	84.70	48.34	29.34	25.24

1μM	0.5μM	0.25μM	0.13μM	0.063μM	0.031μM	0.0156μM	0.0078μM
329.8	250.7	177.1	123.4	77.58	49.06	32.80	25.31

E

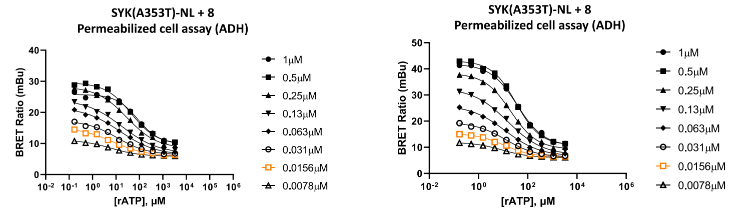

1μM	0.5μM	0.25μM	0.13μM	0.063μM	0.031μM	0.0156μM	0.0078μM
84.28	45.43	29.35	18.12	14.56	11.10	7.210	6.099

1μM	0.5μM	0.25μM	0.13μM	0.063μM	0.031μM	0.0156μM	0.0078μM
37.55	33.70	20.46	15.50	12.16	12.96	10.97	9.064

F

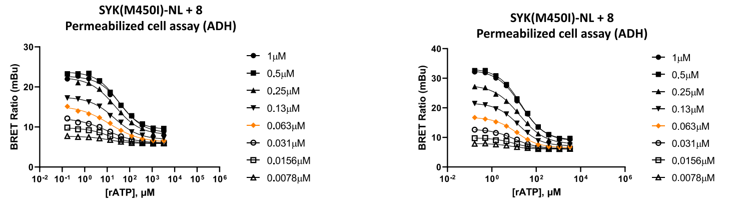

1μM	0.5μM	0.25μM	0.13μM	0.063μM	0.031μM	0.0156μM	0.0078μM
40.84	29.61	22.12	21.43	13.24	9.791	11.05	13.08

1μM	0.5μM	0.25μM	0.13μM	0.063μM	0.031μM	0.0156μM	0.0078μM
20.80	20.75	17.23	15.50	12.92	10.23	10.06	7.824

Supplementary Figure 4. SYK NanoBRET assay with titrations of tracer **8** in cells permeabilized with digitonin. An 11-point dose–response study of ATP from a top concentration of 10 mM was used to compete away the BRET signal. Two biological replicates (left and right) and the results plotted in

GraphPad Prism using a log(inhibitor) vs. response (three parameter) fit. IC_{50} data are reported in µM. (**A**) SYK(WT)-NL (**B**) SYK(S550Y)-NL (**C**) SYK(S550F)-NL (**D**) SYK(P342T)-NL (**E**) SYK(A353T)-NL (**F**) SYK(M450I)-NL.

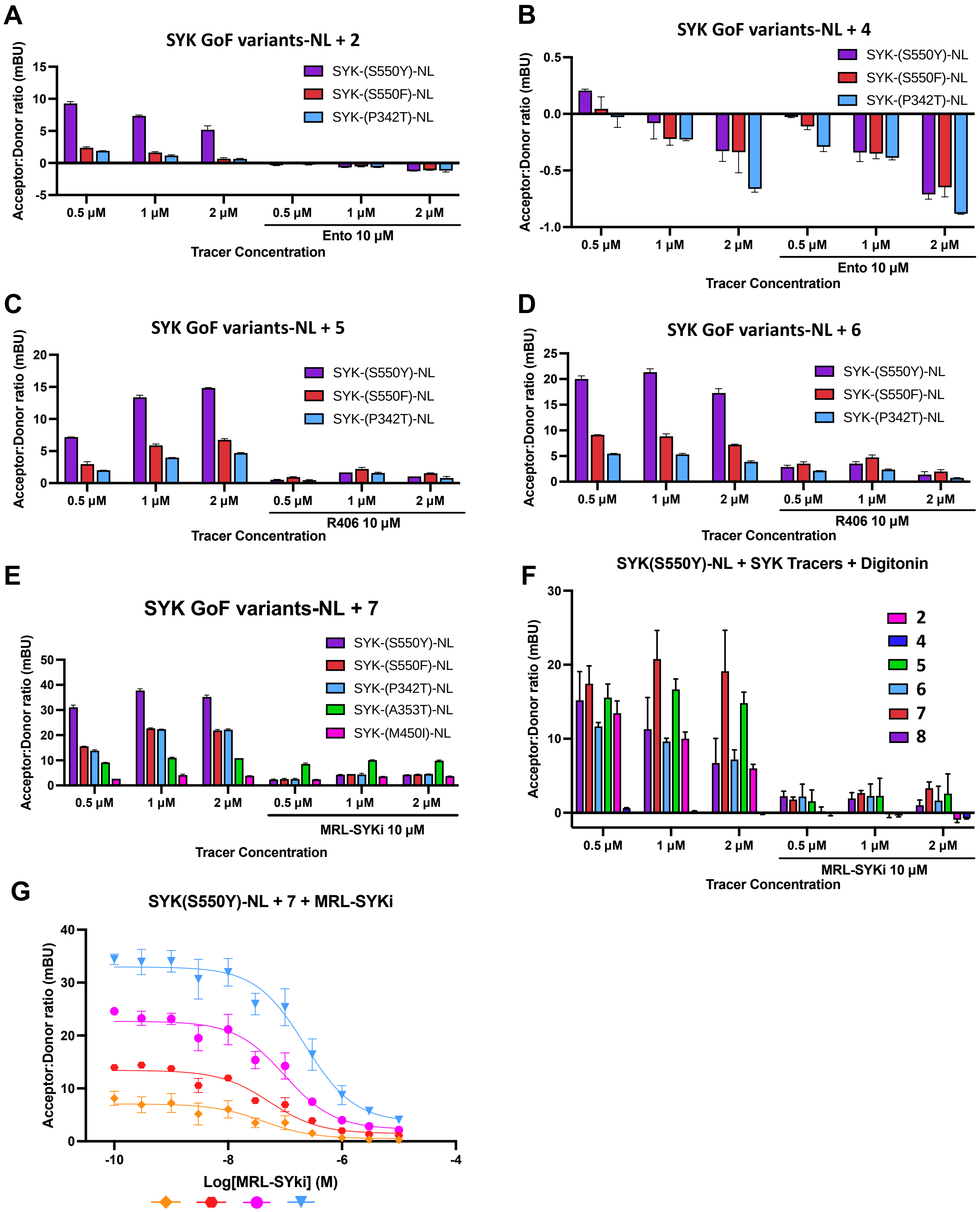

[Tracer] (nM)	125	250	500	1000
IC_{50} (nM)	41	54	98	220

Supplementary Figure 5. SYK NanoBRET tracers **2** (**A**), **5** (**C**), **6** (**D**), **7** (**E**) generated BRET signal (mBU) with SYK GoF variants in the NanoBRET assay that can be competed away with SYK inhibitors. BRET signal is greater with SYK(S550Y) than SYK(S550F) and least with SYK(P342T). (**B**) Tracer **4** does not generate a BRET signal with any GoF variants, which correlates with its inability to bind to SYK in enzymatic inhibition assays. (**F**) Tracers **2**, **5**, **6**, **7**, and **8** all generate a BRET signal with SYK(S550Y) + digitonin. The mBU are decreased compared to intact cells. (**G**) Tracer titrations with **8** and SYK(S550Y)-NL. Data are reported in biological duplicate ± standard deviation (**A**–**E**, **G**). Data are reported in biological duplicates each with two technical replicates ± standard deviation (**F**).

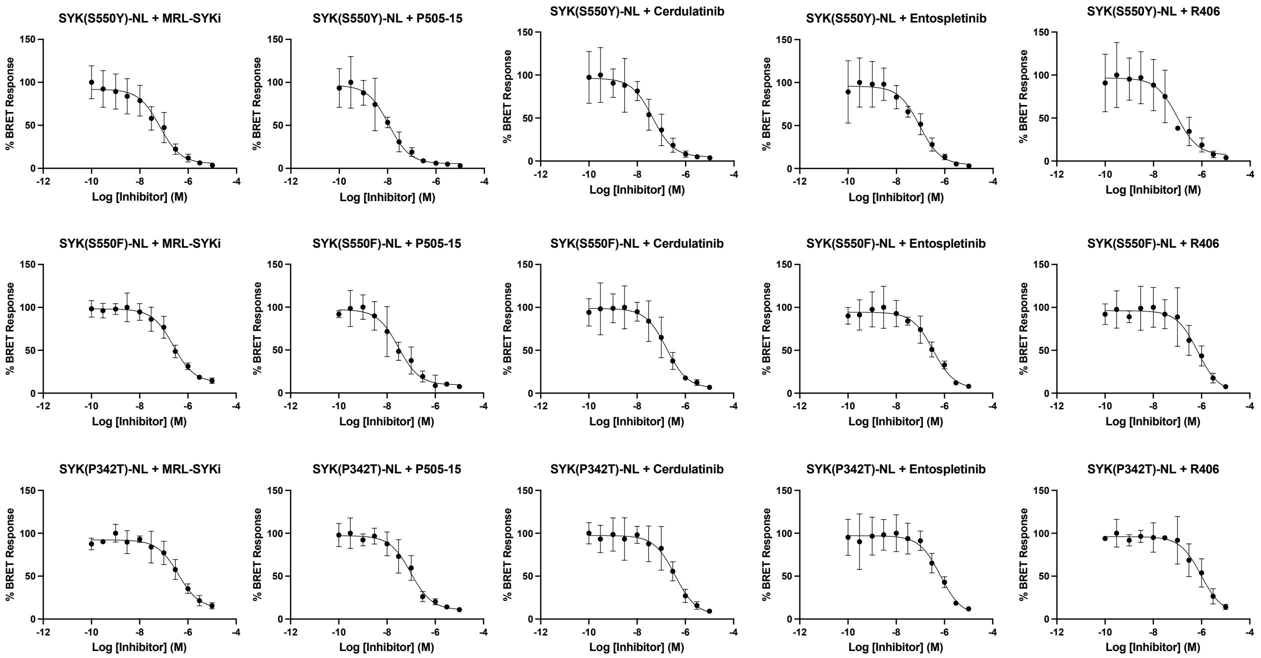

Supplementary Figure 6. SYK inhibitor dose–response curves with SYK(GoF)-NL variants. Data are reported as three biological replicates ± standard deviation and plotted in GraphPad Prism using a log(inhibitor) vs. response (three parameter) fit.

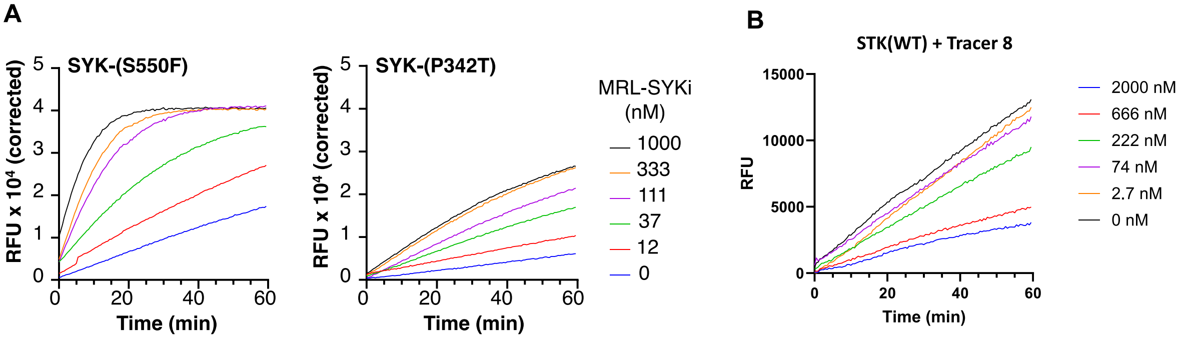

Supplementary Figure 7. SYK PhosphoSens Assay with SYK SOX-peptide substrate. (**A**) Raw data for the dose–response curves of MRL-SYKi with SYK(S550F) and SYK(P342T) in Figure 5D within the main text. (**B**) Dose–response evaluation of tracer **8** with SYK(WT) indicating inhibition of SYK catalytic activity.

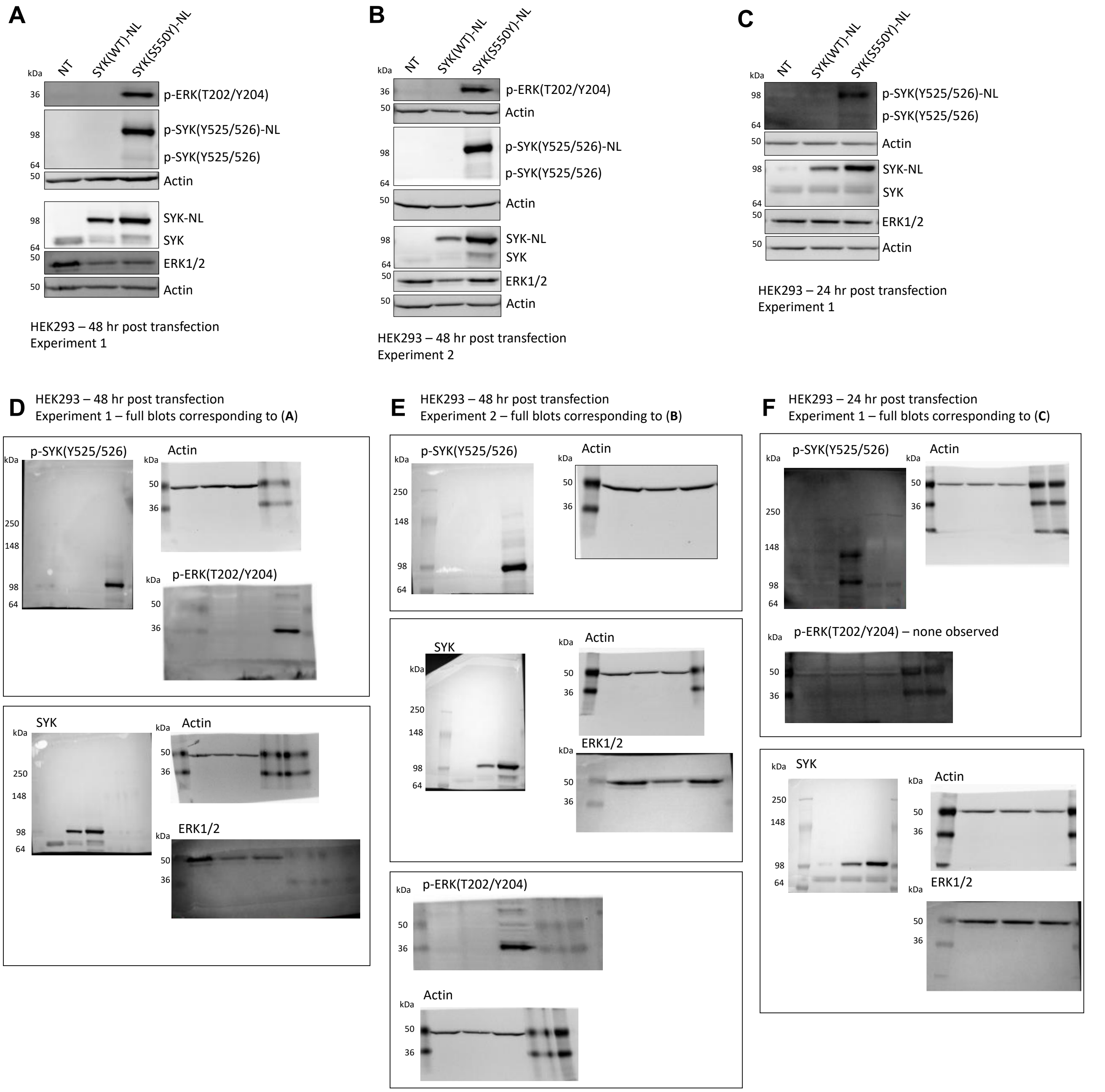

Supplementary Figure 8. SYK and pSYK(Y525/526) expression levels 48 hr (**A**–**B**) and 24 hr (**C**) post-transfection of SYK(WT)-NL and SYK(S550Y)-NL in HEK293 cells. pERK(T202/Y204) levels are increased after 48 hr, but not after 24 hr. (**D**–**E**) Full blots corresponding to the western blots in (**A**–**B**). (**F**) Full blots corresponding to the western blots in (**A**–**C**).

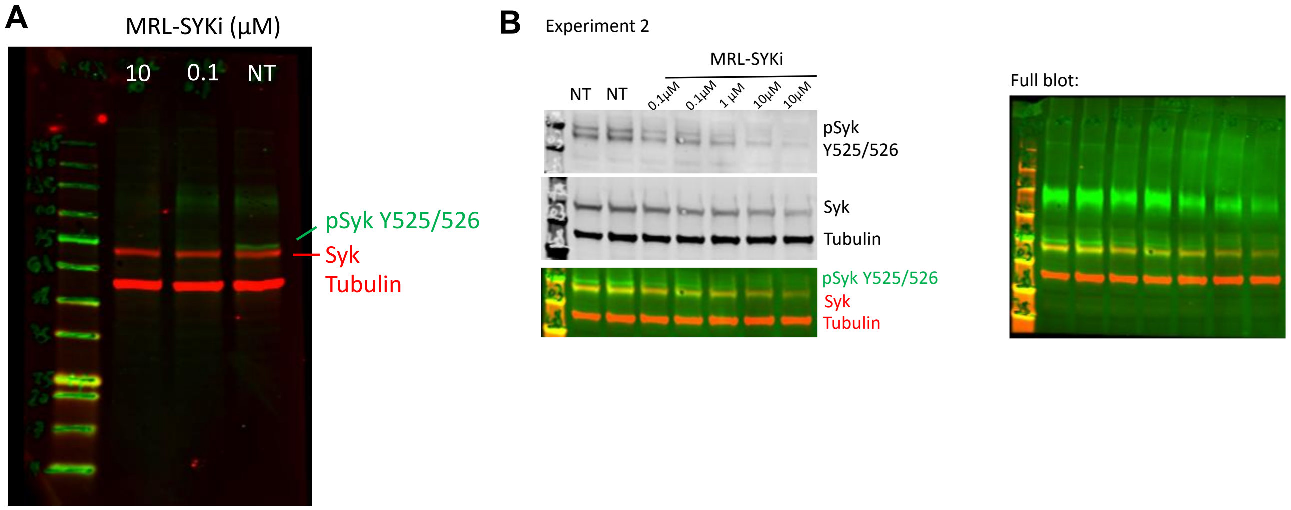

Supplementary Figure 9. Phosphorylation levels at SYK(Y525/526) decrease upon MRL-SYKi inhibitor treatment after 20 hrs at 100 nM and 10 µM in a stably expressing SYK(S550Y) SW480 cell line. (**A**) Full western blot corresponding to the western blot included in Figure 5E in the main text (biological replicate number 1). (**B**) Biological replicate number 2 of MRL-SYKi inhibitor treatment.

2 Chemistry Experimental

2.1 General Information for Chemical Synthesis

Reagents were obtained from verified suppliers and employed without purification. Temperatures are in degrees Celsius (°C); solvent was removed using a rotary evaporator; thin layer chromatography and LC–MS were used to monitor reaction progress. ^{1}H NMR and additional analytical data was collected for intermediates and final compounds to verify their identity and evaluate their purity. ^{1}H and ^{13}C NMR spectra were obtained in DMSO-d_6, MeOD-d_4, or $CDCl_3$ and recorded using Bruker spectrometers. Magnet strength is listed in each experimental write-up along with peak positions in parts per million (ppm). Peaks in NMR spectra are calibrated versus the shift of the indicated deuterated solvent and coupling constants (J values) are reported in hertz (Hz). Peak multiplicities are included as follows: singlet (s), doublet (d), doublet of doublets (dd), triplet (t), pentet (p), and multiplet (m). The purity of all final compounds was assessed via HPLC.

2.2 Abbreviations

DIPEA (*N*-ethyl-*N*-isopropylpropan-2-amine); DMF (*N*,*N*-dimethylformamide), MeOH (methanol), EtOH (ethanol), Ar (argon), TBTU (2-(1*H*-benzo[*d*][1,2,3]triazol-1-yl)-1,1,3,3-tetramethyluronium tetrafluoroborate), rt (room temperature), H_2O (water), TFA (trifluoroacetic acid), THF (tetrahydrofuran).

2.3 Chemistry Schemes

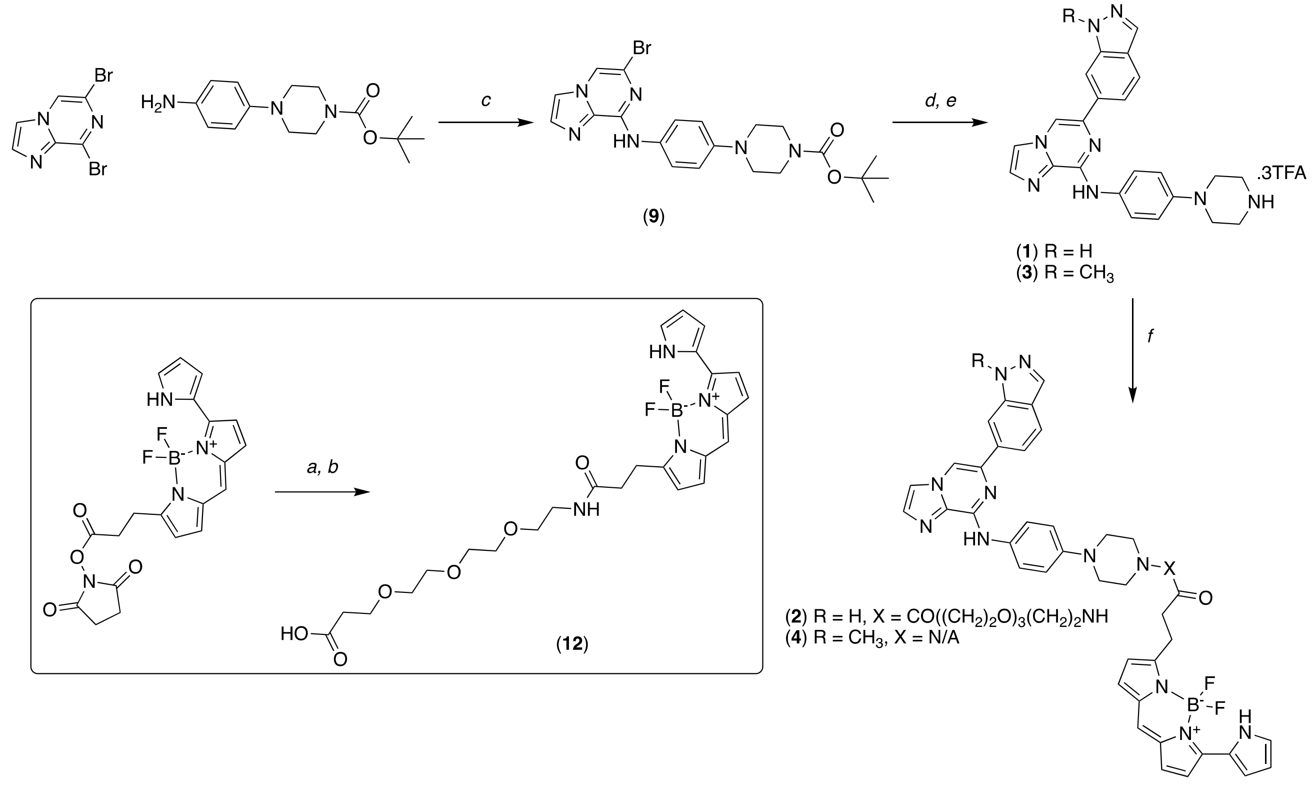

Scheme 1. Synthesis of NanoBRET tracers **2** and **4**. Reagents and conditions: a) *tert*-butyl 3-(2-(2-(2-aminoethoxy)ethoxy)ethoxy)propanoate, 2,5-dioxopyrrolidin-1-yl 3-(5,5-difluoro-7-(1*H*-pyrrol-2-yl)-5*H*-5λ[4],6λ[4]-dipyrrolo[1,2-c:2',1'-*f*][1,3,2]diazaborinin-3-yl)propanoate, DIPEA, DMF. b) TFA, CH_2Cl_2, rt, 16 hrs. c) (**9**): 6,8-dibromoimidazo[1,2-*a*]pyrazine, *tert*-butyl 4-(4-aminophenyl)piperazine-1-carboxylate, DIPEA, propan-2-ol, 85 °C, 16 hrs. d) (1*H*-indazol-6-yl)boronic acid or (1-methyl-1*H*-indazol-6-yl)boronic acid , **9**, Na_2CO_3, $PdCl_2$(dppf), H_2O, 1,4-dioxane (0.25 M), 100 °C, N_2, 16 hrs. e) TFA, CH_2Cl_2, rt, 16 hrs. f) (**2**): **12**, **1**, DIPEA, TBTU, DMF, rt, 16 hrs. (**4**): 2,5-dioxopyrrolidin-1-yl 3-(5,5-difluoro-7-(1*H*-pyrrol-2-yl)-5*H*-5λ[4],6λ[4]-dipyrrolo[1,2-*c*:2',1'-*f*][1,3,2]diazaborinin-3-yl)propanoate, **3**, DIPEA, DMF, rt, 10 mins.

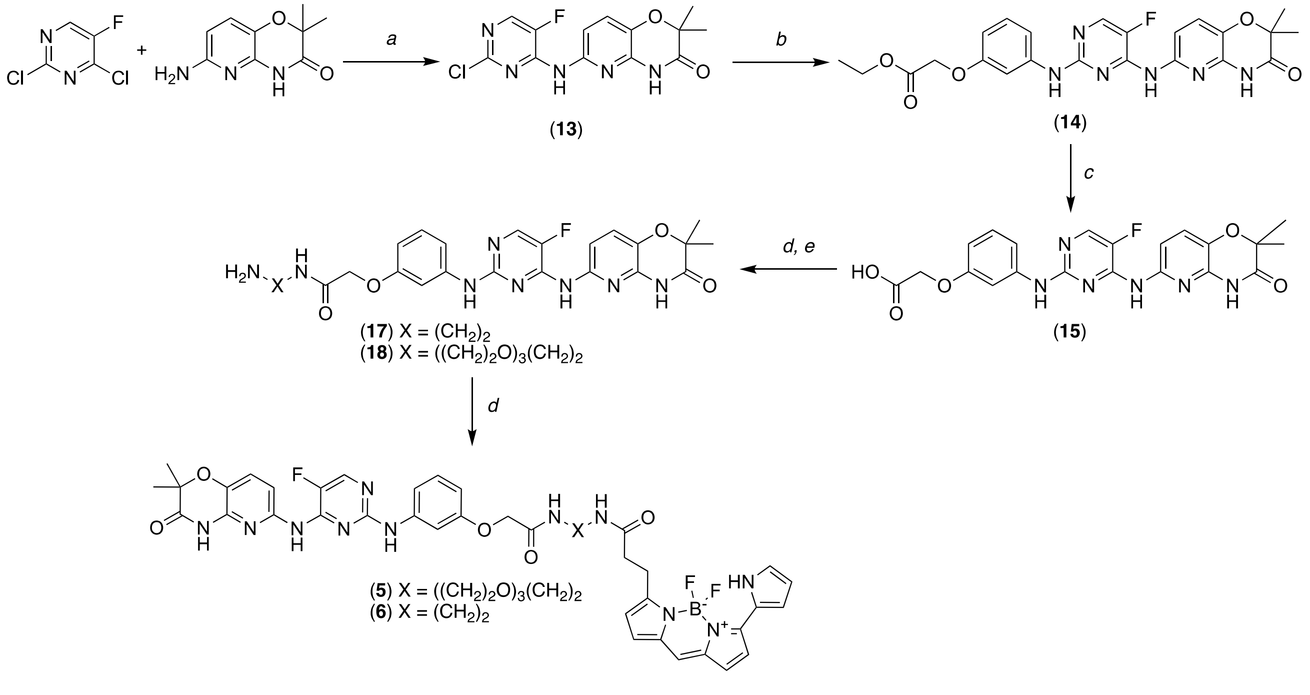

Scheme 2. Synthesis of NanoBRET tracers **5** and **6**. Reagents and conditions: a) 2,4-dichloro-5-fluoropyrimidine, 6-amino-2,2-dimethyl-2*H*-pyrido[3,2-*b*][1,4]oxazin-3(4*H*)-one, MeOH, H_2O, 80 °C, 16 hrs. b) ethyl 2-(3-aminophenoxy)acetate, **13**, EtOH, Ar, 80 °C, 16 hrs. c) **14**, LiOH, THF, H_2O, 50 ºC, 16 hrs. d) (**17**): **15**, DIPEA, TBTU, *tert*-butyl (2-aminoethyl)carbamate, DMF, 72 hrs. (**18**): **15**, DIPEA, TBTU, *tert*-butyl (2-(2-(2-(2-aminoethoxy)ethoxy)ethoxy)ethyl)carbamate, DMF, 16 hrs. e) TFA, CH_2Cl_2, rt, 16 hrs. d) **17** or **18**, 2,5-dioxopyrrolidin-1-yl 3-(5,5-difluoro-7-(1*H*-pyrrol-2-yl)-5*H*-5λ^4,6λ^4-dipyrrolo[1,2-*c*:2',1'-*f*][1,3,2]diazaborinin-3-yl)propanoate, DIPEA, DMF, rt, 30 mins.

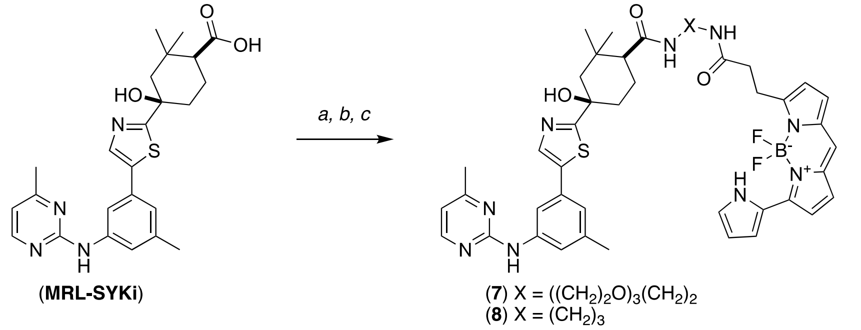

Scheme 3. Synthesis of NanoBRET tracers **7** and **8**. Reagents and conditions: a) (**7**): MRL-SYKi, *tert*-butyl (2-(2-(2-(2-aminoethoxy)ethoxy)ethoxy)ethyl)carbamate, DIPEA, HATU, DMF, rt,16 hrs. b) TFA, CH_2Cl_2, rt, 1 hr. c) (1*S*,4*R*)-*N*-(2-(2-(2-(2-aminoethoxy)ethoxy)ethoxy)ethyl)-4-hydroxy-2,2-dimethyl-4-(5-(3-methyl-5-((4-methylpyrimidin-2-yl)amino)phenyl)thiazol-2-yl)cyclohexane-1-carboxamide 2,2,2-trifluoroacetate, 2,5-dioxopyrrolidin-1-yl 3-(5,5-difluoro-7-(1*H*-pyrrol-2-yl)-5*H*-5λ4,6λ4-dipyrrolo[1,2-*c*:2',1'-*f*][1,3,2]diazaborinin-3-yl)propanoate, DIPEA, DMF, rt, 30 mins. (**8**): a) MRL-SYKi *tert*-butyl (3-aminopropyl)carbamate, DIPEA, HATU, DMF, rt,16 hrs. b) TFA, CH_2Cl_2, rt, 1 hr. c) (1*S*,4*R*)-*N*-(3-aminopropyl)-4-hydroxy-2,2-dimethyl-4-(5-(3-methyl-5-((4-methylpyrimidin-2-yl)amino)phenyl)thiazol-2-yl)cyclohexane-1-carboxamide 2,2,2-trifluoroacetate, 2,5-dioxopyrrolidin-1-yl 3-(5,5-difluoro-7-(1*H*-pyrrol-2-yl)-5*H*-5λ4,6λ4-dipyrrolo[1,2-*c*:2',1'-*f*][1,3,2]diazaborinin-3-yl)propanoate, DIPEA, DMF, rt, 30 mins.

2.4 Chemistry Experimental Data

***Tert*-butyl 4-(4-((6-bromoimidazo[1,2-*a*]pyrazin-8-yl)amino)phenyl)piperazine-1-carboxylate (9)**

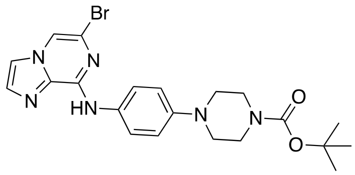

To a flask was added 6,8-dibromoimidazo[1,2-*a*]pyrazine (250 mg, 1.00 equiv., 903 µmol), *tert*-butyl 4-(4-aminophenyl)piperazine-1-carboxylate (263 mg, 1.05 equiv., 948 µmol), and DIPEA (236 µL, 1.50 equiv., 1.35 mmol) in propan-2-ol (3.61 mL, 0.25 molar, 1.00 equiv., 903 µmol) and the reaction was heated to 85 °C for 16 hrs. The reaction was concentrated *in vacuo* and purified via column chromatography (silica, 0-100% EtOAc in hexane) to yield the desired product as a yellow solid (218 mg, 51%). ^{1}H NMR (400 MHz, $CDCl_3$) δ 9.25 (s, 1H), 7.86 (d, *J* = 8.9 Hz, 2H), 7.74 (s, 1H), 7.66 – 7.56 (m, 2H), 7.14 (s, 2H), 3.70 (s, 4H), 3.18 (s, 4H), 1.48 (s, 9H). LCMS calculated for $C_{21}H_{26}BrN_6O_2$ $[M + H]^+$: 473.12. Found: 473.08.

***Tert*-butyl 4-(4-((6-(1*H*-indazol-6-yl)imidazo[1,2-*a*]pyrazin-8-yl)amino)phenyl)piperazine-1-carboxylate (10)**

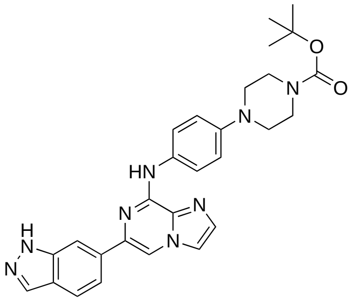

To a microwave vial was added (1*H*-indazol-6-yl)boronic acid (112 mg, 1.5 equiv., 690 µmol), *tert*-butyl 4-(4-((6-bromoimidazo[1,2-*a*]pyrazin-8-yl)amino)phenyl)piperazine-1-carboxylate (218 mg, 1.0 equiv., 460 µmol), Na_2CO_3 (73 mg, 1.5 equiv., 690 µmol), and $PdCl_2$(dppf) (34 mg, 0.1 equiv., 46 µmol) in H_2O (460 µL) and 1,4-dioxane (1.84 mL, 0.25 M) and the reaction was heated to 100 °C under N_2 for 16 hrs. The reaction was cooled to rt, filtered through celite, and concentrated *in vacuo*. The crude material was purified by column chromatography (silica, 0-10% MeOH in CH_2Cl_2) to yield the desired product as a yellow solid (141 mg, 60%). ^{1}H NMR (400 MHz, DMSO) δ 13.18 (s, 1H), 9.54 (s, 1H), 8.67 (s, 1H), 8.18 (s, 1H), 8.09 (s, 1H), 8.03 (d, *J* = 9.1 Hz, 2H), 7.99 (d, *J* = 1.0 Hz, 1H), 7.84 (d, *J* = 8.6 Hz, 1H), 7.72 (dd, *J* = 8.6, 1.3 Hz, 1H), 7.64 (d, *J* = 1.0 Hz, 1H), 7.02 (d, *J* = 9.1 Hz, 2H), 3.53 – 3.45 (m, 4H), 3.12 – 3.05 (m, 4H), 1.43 (s, 9H). ^{13}C NMR (214 MHz, DMSO) δ 153.91, 146.46, 144.83, 140.51, 136.46, 135.18, 133.47, 132.79, 132.31, 132.14, 122.58, 121.15, 120.67, 118.31, 116.47, 108.72, 107.12, 79.00, 49.18, 28.09.

6-(1-methyl-1*H*-indazol-6-yl)-*N*-(4-(piperazin-1-yl)phenyl)imidazo[1,2-*a*]pyrazin-8-amine (3)

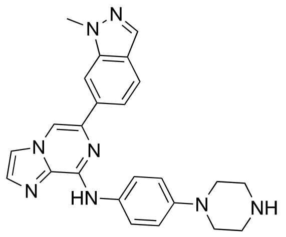

To a microwave vial was added *tert*-butyl 4-(4-((6-bromoimidazo[1,2-*a*]pyrazin-8-yl)amino)phenyl)piperazine-1-carboxylate (50 mg, 1.0 equiv., 0.11 mmol), (1-methyl-1*H*-indazol-6-yl)boronic acid (37 mg, 2.0 equiv., 0.21 mmol), Na_2CO_3 (25 mg, 2.2 equiv., 0.23 mmol), 1,4-dioxane (0.74 mL) and H_2O (0.3 mL). The solution was degassed with nitrogen for 5 mins before $Pd(PPh_3)_4$ (12 mg, 0.1 equiv., 11 μmol) was added. The reaction was heated at 100˚C for 19 hrs. The reaction was cooled, dissolved in EtOAc, and filtered *in vacuo* through celite. The crude material was purified by column chromatography (silica, 0-100% EtOAc in hexane) to yield *tert*-butyl 4-(4-((6-(1-methyl-1*H*-indazol-6-yl)imidazo[1,2-*a*]pyrazin-8-yl)amino)phenyl)piperazine-1-carboxylate, which was used in the next step. To a flask was added *tert*-butyl 4-(4-((6-(1-methyl-1*H*-indazol-6-yl)imidazo[1,2-*a*]pyrazin-8-yl)amino)phenyl)piperazine-1-carboxylate (29 mg, 1.0 equiv., 55 μmol) with 20% TFA in CH_2Cl_2. The reaction was stirred at rt for 36 hrs, concentrated *in vacuo*, and purified by preparative-HPLC (10-100% MeOH in H_2O (+ 0.01% TFA) to yield the desired product as a white solid. ^1H NMR (400 MHz, MeOD-*d*4) δ 8.52 (d, *J* = 1.6 Hz, 1H), 8.11 (s, 1H), 8.04 – 7.96 (m, 2H), 7.91 – 7.87 (m, 2H), 7.83 (d, *J* = 1.5 Hz, 1H), 7.78 – 7.75 (m, 1H), 7.73 – 7.70 (m, 1H), 7.10 – 7.02 (m, 2H), 4.06 (d, *J* = 1.3 Hz, 3H), 3.39 (s, 8H). ^{13}C NMR (214 MHz, MeOD-*d*4) δ 147.69, 144.85, 141.73, 140.52, 136.74, 134.85, 133.72, 130.62, 125.27, 122.29, 122.22, 118.74, 117.96, 116.72, 115.39, 109.94, 108.00, 54.78, 48.46, 44.93, 35.58. LCMS calculated for $C_{24}H_{25}N_8$ $[M + H]^+$: 425.21. Found: 425.53.

3-(5,5-difluoro-7-(1*H*-pyrrol-2-yl)-5*H*-5λ⁴,6λ⁴-dipyrrolo[1,2-*c*:2',1'-*f*][1,3,2]diazaborinin-3-yl)-1-(4-(4-((6-(1-methyl-1*H*-indazol-6-yl)imidazo[1,2-*a*]pyrazin-8-yl)amino)phenyl)piperazin-1-yl)propan-1-one (4)

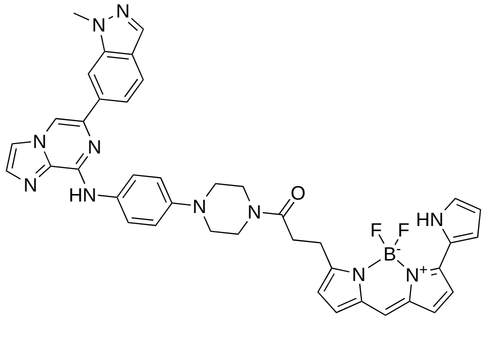

To a flask was added 2,5-dioxopyrrolidin-1-yl 3-(5,5-difluoro-7-(1*H*-pyrrol-2-yl)-5*H*-5λ^4,6λ^4-dipyrrolo[1,2-*c*:2',1'-*f*][1,3,2]diazaborinin-3-yl)propanoate (5 mg, 1.0 equiv., 0.01 mmol), 6-(1-methyl-1*H*-indazol-6-yl)-*N*-(4-(piperazin-1-yl)phenyl)imidazo[1,2-*a*]pyrazin-8-amine (5 mg, 1.0 equiv, 0.01 mmol), and DIPEA (10 μL, 6.0 equiv., 0.06 mmol) in DMF (100 μL, 0.1M). The reaction was stirred at rt for 10 mins. The reaction was concentrated *in vacuo* and purified by preparative-HPLC (10-100% MeOH in H_2O (+ 0.01% TFA) to yield the desired product as a purple solid (0.9 mg, 10%). ^1H NMR (400 MHz, CD_3OD) δ 8.55 (s, 1H), 8.19 (s, 1H), 8.02 (s, 1H), 8.00 – 7.93 (m, 3H), 7.84 – 7.76 (m,

2H), 7.75 (s, 1H), 7.23 (s, 1H), 7.21 – 7.16 (m, 3H), 7.14 (d, *J* = 8.9 Hz, 2H), 6.99 (d, *J* = 4.6 Hz, 1H), 6.92 (d, *J* = 4.0 Hz, 1H), 6.36 (d, *J* = 3.9 Hz, 1H), 6.34 – 6.31 (m, 1H), 4.09 (s, 3H), 3.85 – 3.80 (m, 2H), 3.77 – 3.72 (m, 2H), 3.24 – 3.20 (m, 2H), 3.19 – 3.25 (m, 2H), 2.95 – 2.84 (m, 2H). LCMS calculated for $C_{40}H_{37}BF_2N_{11}O$ $[M + H]^+$: 736.32. Found: 736.0.

***Tert*-butyl 1-(5,5-difluoro-7-(1*H*-pyrrol-2-yl)-5*H*-5λ⁴,6λ⁴-dipyrrolo[1,2-*c*:2',1'-*f*][1,3,2]diazaborinin-3-yl)-3-oxo-7,10,13-trioxa-4-azahexadecan-16-oate (11)**

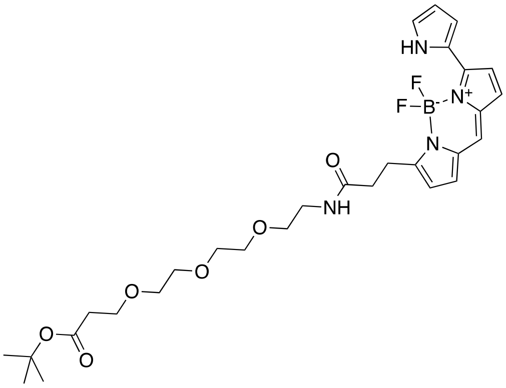

To a flask was added *tert*-butyl 3-(2-(2-(2-aminoethoxy)ethoxy)ethoxy)propanoate (6.5 mg, 1.0 equiv., 23 µmol), 2,5-dioxopyrrolidin-1-yl 3-(5,5-difluoro-7-(1*H*-pyrrol-2-yl)-5*H*-5λ^4,6λ^4-dipyrrolo[1,2-c:2',1'-*f*][1,3,2]diazaborinin-3-yl)propanoate (10 mg, 1.0 equiv., 23 µmol), and DIPEA (25 µL, 6.0 equiv., 0.14 mmol) in DMF (0.47 mL, 0.05 M). The reaction was concentrated *in vacuo* and the crude material was purified by column chromatography (silica, 0-10% MeOH in CH_2Cl_2) to yield the desired product as a purple solid (14 mg, 100 %). ^{1}H NMR (400 MHz, MeOD-d_4) δ 7.99 (s, 1H), 7.24 (dd, *J* = 16.1, 6.9 Hz, 4H), 7.03 (d, *J* = 4.7 Hz, 1H), 6.93 (s, 1H), 6.39 – 6.34 (m, 1H), 3.74 – 3.51 (m, 14H), 3.40 (d, *J* = 5.6 Hz, 2H), 2.71 – 2.63 (m, 2H), 2.53 – 2.41 (m, 2H), 1.45 (dd, *J* = 10.4, 1.3 Hz, 9H).

1-(5,5-difluoro-7-(1*H*-pyrrol-2-yl)-5*H*-5λ⁴,6λ⁴-dipyrrolo[1,2-*c*:2',1'-*f*][1,3,2]diazaborinin-3-yl)-3-oxo-7,10,13-trioxa-4-azahexadecan-16-oic acid (12)

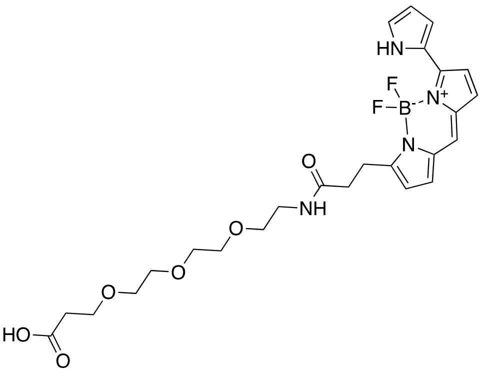

To a flask was added *tert*-butyl 1-(5,5-difluoro-7-(1*H*-pyrrol-2-yl)-5*H*-5λ^4,6λ^4-dipyrrolo[1,2-*c*:2',1'-*f*][1,3,2]diazaborinin-3-yl)-3-oxo-7,10,13-trioxa-4-azahexadecan-16-oate (**11**) (14 mg, 1.0 equiv., 24 µmol) in 20% TFA in CH_2Cl_2 (1 mL) and the reaction stirred at rt for 16 hrs. The reaction was concentrated *in vacuo* and used in the next step without further purification (7.1 mg).

_N_-(2-(2-(2-(3-(4-(4-((6-(1_H_-indazol-6-yl)imidazo[1,2-_a_]pyrazin-8-yl)amino)phenyl)piperazin-1-yl)-3-oxopropoxy)ethoxy)ethoxy)ethyl)-3-(5,5-difluoro-7-(1_H_-pyrrol-2-yl)-5_H_-5λ^4,6λ^4-dipyrrolo[1,2-_c_:2',1'-_f_][1,3,2]diazaborinin-3-yl)propenamide (2)

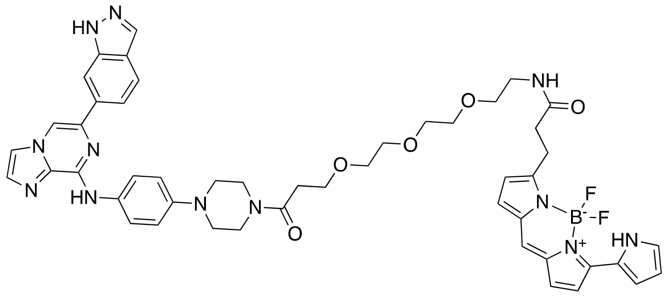

To a flask was added 1-(5,5-difluoro-7-(1*H*-pyrrol-2-yl)-5*H*-5λ^4,6λ^4-dipyrrolo[1,2-*c*:2',1'-*f*][1,3,2]diazaborinin-3-yl)-3-oxo-7,10,13-trioxa-4-azahexadecan-16-oic acid (**12**) (7.1 mg, 1.0 equiv., 13 µmol), DIPEA (7.4 µL, 3.2 equiv., 43 µmol), and TBTU (6.4 mg, 1.5 equiv., 20 µmol) in DMF (130 µL, 0.1M) and the reaction stirred at rt for 30 mins. (**1**) (10 mg, 1.0 equiv., 13 µmol) was added and the reaction was stirred at rt for 16 hrs. The reaction was concentrated and purified via preparative-HPLC (10-100% MeOH in H_2O (+ 0.01% TFA) to yield the desired product as a purple solid (0.5 mg, 3%). ^{1}H NMR (400 MHz, $CDCl_3$) δ 10.28 (s, 1H), 9.98 (s, 1H), 8.25 (s, 1H), 8.11 (s, 1H), 8.00 (s, 1H), 7.92 (d, *J* = 9.0 Hz, 2H), 7.81 (d, *J* = 8.5 Hz, 1H), 7.70 (d, *J* = 1.7 Hz, 1H), 7.62 (d, *J* = 1.7 Hz, 1H), 7.52 (d, *J* = 4.5 Hz, 1H), 7.12 (d, *J* = 8.8 Hz, 2H), 7.05 (s, 1H), 6.95 (d, *J* = 4.6 Hz, 1H), 6.91 – 6.86 (s, 2H), 6.76 (dd, *J* = 7.2, 4.3 Hz, 2H), 6.30 (s, 1H), 6.24 (d, *J* = 4.0 Hz, 1H), 3.86 (s, 2H), 3.81 – 3.72 (m, 4H), 3.58 (d, *J* = 13.0 Hz, 8H), 3.52 (t, *J* = 4.9 Hz, 2H), 3.44 (d, *J* = 5.1 Hz, 2H), 3.29 (t, *J* = 7.6 Hz, 2H), 3.24 (s, 2H), 3.19 (s, 2H), 2.65 – 2.57 (m, 4H). LCMS calculated for $C_{48}H_{52}BF_2N_{12}O_5$ [M + H]$^+$: 925.42. Found: 925.30

6-((2-chloro-5-fluoropyrimidin-4-yl)amino)-2,2-dimethyl-2_H_-pyrido[3,2-_b_][1,4]oxazin-3(4_H_)-one (13)

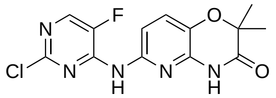

To a flask was added 2,4-dichloro-5-fluoropyrimidine (432 mg, 1.0 equiv., 2.6 mmol), 6-amino-2,2-dimethyl-2*H*-pyrido[3,2-*b*][1,4]oxazin-3(4*H*)-one (500 mg, 1.0 equiv., 2.6 mmol), MeOH (4.31 mL), and H_2O (933 µL) and the reaction was heated at 80 °C for 16 hrs. The reaction was cooled and the precipitate was filtered under vacuum with MeOH to yield the desired product as a white solid (543 mg, 64.9 %). ^{1}H NMR (400 MHz, DMSO) δ 11.15 (s, 1H), 10.11 (s, 1H), 8.36 (d, *J* = 3.3 Hz, 1H), 7.51 – 7.39 (m, 2H), 1.43 (s, 6H). ^{13}C NMR (214 MHz, DMSO) δ 169.62, 152.83, 150.90, 150.85, 145.68, 144.46, 143.06, 142.46, 142.36, 125.88, 111.38, 77.99, 23.54. LCMS calculated for $C_{13}H_{12}ClFN_5O_2$ [M + H]$^+$: 324.06. Found: 324.13.

Ethyl 2-(3-((4-((2,2-dimethyl-3-oxo-3,4-dihydro-2_H_-pyrido[3,2-_b_][1,4]oxazin-6-yl)amino)-5-fluoropyrimidin-2-yl)amino)phenoxy)acetate (14)

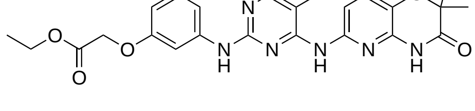

To a microwave vial was added ethyl 2-(3-aminophenoxy)acetate (110 mg, 1.2 equiv., 563 µmol), 6-((2-chloro-5-fluoropyrimidin-4-yl)amino)-2,2-dimethyl-2*H*-pyrido[3,2-*b*][1,4]oxazin-3(4*H*)-one (152 mg, 1.0 equiv., 470 µmol), and EtOH (2 mL). The reaction was degassed with Ar and heated to 80 °C for 16 hrs. The reaction was cooled and the precipitate filtered with ethanol to yield the desired product as a white solid (132 mg, 58%). ^{1}H NMR (400 MHz, DMSO) δ 11.09 (s, 1H), 9.26 (s, 2H), 8.19 – 8.12 (m, 1H), 7.65 – 7.60 (m, 1H), 7.43 – 7.33 (m, 2H), 7.31 – 7.26 (m, 1H), 7.14 – 7.06 (m, 1H), 6.48 – 6.43 (m, 1H), 4.72 – 4.64 (m, 2H), 4.22 – 4.13 (m, 2H), 1.48 – 1.40 (m, 6H), 1.27 – 1.17 (m, 3H). ^{13}C NMR (214 MHz, DMSO) δ 169.69, 168.76, 157.85, 155.27, 149.17, 144.29, 141.97, 140.04, 139.87, 133.81, 129.05, 125.75, 111.79, 110.85, 106.28, 105.28, 77.85, 64.60, 60.59, 23.47, 14.05. LCMS calculated for $C_{23}H_{24}FN_6O_5$ [M + H]$^+$: 483.17. Found: 482.87.

2-(3-((4-((2,2-dimethyl-3-oxo-3,4-dihydro-2*H*-pyrido[3,2-*b*][1,4]oxazin-6-yl)amino)-5-fluoropyrimidin-2-yl)amino)phenoxy)acetic acid (15)

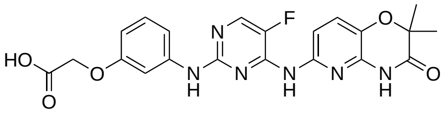

To a flask was added ethyl 2-(3-((4-((2,2-dimethyl-3-oxo-3,4-dihydro-2*H*-pyrido[3,2-*b*][1,4]oxazin-6-yl)amino)-5-fluoropyrimidin-2-yl)amino)phenoxy)acetate (128 mg, 265 µmol, 1.0 equiv.), and LiOH (10 mg, 398 µmol, 1.5 equiv.) in THF (1 mL) and H_2O (239 µL, 50.0 equiv.) and the reaction stirred at 50 ºC for 16 hrs. The reaction was cooled, and HCl (1M) was added. The organics were extracted with EtOAc, washed with brine, and dried with sodium sulfate to yield the desired product as a white solid (66 mg, 55%). ^{1}H NMR (400 MHz, DMSO) δ 11.08 (s, 1H), 9.29 (d, *J* = 9.1 Hz, 2H), 8.15 (d, *J* = 3.5 Hz, 1H), 7.63 (d, *J* = 8.5 Hz, 1H), 7.40 (d, *J* = 8.5 Hz, 1H), 7.34 – 7.25 (m, 2H), 7.10 (t, *J* = 8.1 Hz, 1H), 6.49 – 6.41 (m, 1H), 4.58 (s, 2H), 1.43 (s, 6H). LCMS calculated for $C_{21}H_{20}FN_6O_5$ [M + H]$^+$: 455.14. Found: 455.08.

***Tert*-butyl (2-(2-(3-((4-((2,2-dimethyl-3-oxo-3,4-dihydro-2*H*-pyrido[3,2-*b*][1,4]oxazin-6-yl)amino)-5-fluoropyrimidin-2-yl)amino)phenoxy)acetamido)ethyl)carbamate (16)**

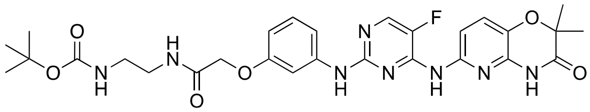

To a flask was added 2-(3-((4-((2,2-dimethyl-3-oxo-3,4-dihydro-2*H*-pyrido[3,2-*b*][1,4]oxazin-6-yl)amino)-5-fluoropyrimidin-2-yl)amino)phenoxy)acetic acid (25 mg, 54 µmol, 1.0 equiv.), DIPEA (30 µL, 173 µmol, 3.2 equiv.), and TBTU (23 mg, 70 µmol, 1.3 equiv.) in DMF (540 µL, 0.1 M). The reaction was allowed to stir at rt for 20 mins followed by addition of *tert*-butyl (2-aminoethyl)carbamate (9 mg, 54 µmol, 1.0 equiv.). The reaction was stirred at rt for 72 hrs. The crude material was concentrated *in vacuo* and purified via column chromatography (silica, 0-20% MeOH in CH_2Cl_2) to yield the desired product as a white solid (16 mg, 51%). ^{1}H NMR (400 MHz, DMSO) δ 11.07 (s, 1H), 9.27 (s, 1H), 9.21 (s, 1H), 8.14 (d, *J* = 3.4 Hz, 1H), 7.64 (d, *J* = 8.5 Hz, 1H), 7.41 – 7.35 (m, 2H), 7.29 (d, *J* = 8.2 Hz, 1H), 7.11 (t, *J* = 8.2 Hz, 1H), 6.49 (dd, *J* = 8.2, 2.4 Hz, 1H), 4.38 (s, 2H), 3.16 (t, *J* = 6.0 Hz, 2H), 3.05 – 2.,99 (m, 2H), 1.43 (s, 6H), 1.37 (s, 9H). LCMS calculated for $C_{28}H_{34}FN_8O_6$ [M + H]$^+$: 597.25. Found: 597.34.

***N*-(2-aminoethyl)-2-(3-((4-((2,2-dimethyl-3-oxo-3,4-dihydro-2*H*-pyrido[3,2-*b*][1,4]oxazin-6-yl)amino)-5-fluoropyrimidin-2-yl)amino)phenoxy)acetamide (17)**

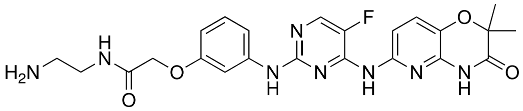

To a flask was added *tert*-butyl (2-(2-(3-((4-((2,2-dimethyl-3-oxo-3,4-dihydro-2*H*-pyrido[3,2-*b*][1,4]oxazin-6-yl)amino)-5-fluoropyrimidin-2-yl)amino)phenoxy)acetamido)ethyl)carbamate (17 mg, 28 µmol, 1.0 equiv.) and TFA (277 µL) in CH_2Cl_2 (1 mL) and the reaction stirred at rt for 16 hrs. The reaction was concentrated *in vacuo* to yield the desired product (7 mg, 41%). ^{1}H NMR (400 MHz, DMSO) δ 11.08 (s, 1H), 9.33 (d, *J* = 5.7 Hz, 2H), 8.20 (t, *J* = 5.9 Hz, 1H), 8.15 (d, *J* = 3.5 Hz, 1H), 7.70 (s, 2H), 7.61 (d, *J* = 8.5 Hz, 1H), 7.41 – 7.35 (m, 2H), 7.29 (d, *J* = 8.2 Hz, 1H), 7.12 (t, *J* = 8.2 Hz, 1H), 6.52 (dd, *J* = 8.2, 2.4 Hz, 1H), 4.42 (s, 2H), 3.38 (q, *J* = 6.2 Hz, 2H), 2.89 (q, *J* = 5.9 Hz, 2H), 1.42 (s, 6H). LCMS calculated for $C_{23}H_{26}FN_8O_6$ [M + H]$^+$: 497.20. Found: 497.23.

3-(5,5-difluoro-7-(1*H*-pyrrol-2-yl)-5*H*-5λ^4,6λ^4-dipyrrolo[1,2-*c*:2',1'-*f*][1,3,2]diazaborinin-3-yl)-*N*-(2-(2-(3-((4-((2,2-dimethyl-3-oxo-3,4-dihydro-2*H*-pyrido[3,2-*b*][1,4]oxazin-6-yl)amino)-5-fluoropyrimidin-2-yl)amino)phenoxy)acetamido)ethyl)propenamide (6)

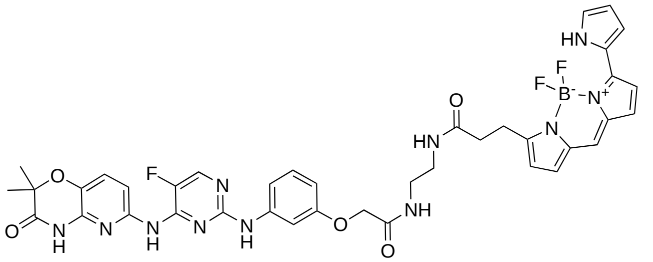

To a flask was added *N*-(2-aminoethyl)-2-(3-((4-((2,2-dimethyl-3-oxo-3,4-dihydro-2*H*-pyrido[3,2-*b*][1,4]oxazin-6-yl)amino)-5-fluoropyrimidin-2-yl)amino)phenoxy)acetamide 2,2,2-trifluoroacetate (7 mg, 0.01 mmol, 1.0 equiv.), 2,5-dioxopyrrolidin-1-yl 3-(5,5-difluoro-7-(1*H*-pyrrol-2-yl)-5*H*-5λ^4,6λ^4-dipyrrolo[1,2-*c*:2',1'-*f*][1,3,2]diazaborinin-3-yl)propanoate (5 mg, 0.01 mmol, 1.0 equiv.), and DIPEA (10 µL, 6.0 equiv.) in DMF (0.1 mL, 0.1 M). The reaction was stirred at rt for 10 mins, concentrated *in vacuo*, and purified by prep-HPLC (10-100% MeOH in H_2O (0.01% TFA) to yield the desired product as a purple solid (3.7 mg, 40 %). ^{1}H NMR (400 MHz, MeOD-*d*$_4$) δ 7.95 (d, *J* = 8.7 Hz, 1H), 7.93 (d, *J* = 3.5 Hz, 1H), 7.41 – 7.39 (m, 1H), 7.23 (d, *J* = 6.3 Hz, 1H), 7.20 (d, *J* = 5.2 Hz, 1H), 7.18 – 7.14 (m, 2H), 7.14 – 7.10 (m, 3H), 6.95 (d, *J* = 4.6 Hz, 1H), 6.83 (d, *J* = 3.9 Hz, 1H), 6.65 – 6.59 (m, 1H), 6.31 (dd, *J* = 3.9, 2.5 Hz, 1H), 6.24 (d, *J* = 4.0 Hz, 1H), 4.49 (s, 2H), 3.42 – 3.35 (m, 2H), 3.22 (t, *J* = 7.8 Hz, 2H), 2.60 – 2.52 (m, 2H), 1.49 (s, 6H). LCMS calculated for $C_{39}H_{38}BF_3N_{11}O_5$ [M + H]$^+$: 808.30. Found: 808.6.

***N*-(2-(2-(2-(2-aminoethoxy)ethoxy)ethoxy)ethyl)-2-(3-((4-((2,2-dimethyl-3-oxo-3,4-dihydro-2*H*-pyrido[3,2-*b*][1,4]oxazin-6-yl)amino)-5-fluoropyrimidin-2-yl)amino)phenoxy)acetamide (18)**

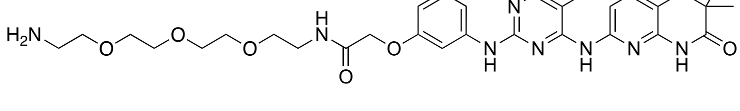

To a flask was added 2-(3-((4-((2,2-dimethyl-3-oxo-3,4-dihydro-2*H*-pyrido[3,2-*b*][1,4]oxazin-6-yl)amino)-5-fluoropyrimidin-2-yl)amino)phenoxy)acetic acid (20 mg, 1.0 equiv., 44 µmol), DIPEA (17 mg, 23 µL, 3.0 equiv., 0.13 mmol), and TBTU (18 mg, 1.3 equiv., 57 µmol) in DMF (0.44 mL, 0.1

M) and the reaction stirred at rt for approx. 5 mins. To this was added *tert*-butyl (2-(2-(2-(2-aminoethoxy)ethoxy)ethoxy)ethyl)carbamate (13 mg, 1.0 equiv., 44 µmol) and the reaction stirred for 16 hrs. The reaction was concentrated *in vacuo* and purified by column chromatography (silica, 0-10% MeOH in CH_2Cl_2) to yield the desired product as a yellow amorphous solid *tert*-butyl (1-(3-((4-((2,2-dimethyl-3-oxo-3,4-dihydro-2*H*-pyrido[3,2-*b*][1,4]oxazin-6-yl)amino)-5-fluoropyrimidin-2-yl)amino)phenoxy)-2-oxo-6,9,12-trioxa-3-azatetradecan-14-yl)carbamate (28.6 mg, 89 %). LCMS calculated for $C_{34}H_{46}FN_8O_9$ $[M + H]^+$: 729.33. Found: 729.25.

To a flask was added *tert*-butyl (1-(3-((4-((2,2-dimethyl-3-oxo-3,4-dihydro-2*H*-pyrido[3,2-*b*][1,4]oxazin-6-yl)amino)-5-fluoropyrimidin-2-yl)amino)phenoxy)-2-oxo-6,9,12-trioxa-3-azatetradecan-14-yl)carbamate (28.6 mg, 39 µmol, 1.0 equiv.) and 20% TFA in CH_2Cl_2 (1 mL), and the reaction stirred at rt for 16 hrs. The reaction was concentrated *in vacuo* to yield the desired product as a yellow solid (25 mg, quant). LCMS calculated for $C_{29}H_{38}FN_8O_7$ $[M + H]^+$: 629.28. Found: 629.17.

3-(5,5-difluoro-7-(1*H*-pyrrol-2-yl)-5*H*-5λ⁴,6λ⁴-dipyrrolo[1,2-*c*:2',1'-*f*][1,3,2]diazaborinin-3-yl)-*N*-(1-(3-((4-((2,2-dimethyl-3-oxo-3,4-dihydro-2*H*-pyrido[3,2-*b*][1,4]oxazin-6-yl)amino)-5-fluoropyrimidin-2-yl)amino)phenoxy)-2-oxo-6,9,12-trioxa-3-azatetradecan-14-yl)propenamide (5)

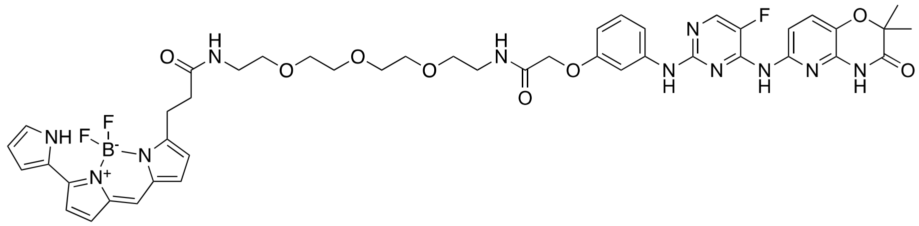

To a flask was added *N*-(2-(2-(2-(2-aminoethoxy)ethoxy)ethoxy)ethyl)-2-(3-((4-((2,2-dimethyl-3-oxo-3,4-dihydro-2*H*-pyrido[3,2-*b*][1,4]oxazin-6-yl)amino)-5-fluoropyrimidin-2-yl)amino)phenoxy)acetamide 2,2,2-trifluoroacetate (5 mg, 7 µmol, 1.0 equiv.), 2,5-dioxopyrrolidin-1-yl 3-(5,5-difluoro-7-(1*H*-pyrrol-2-yl)-5*H*-5λ^4,6λ^4-dipyrrolo[1,2-*c*:2',1'-*f*][1,3,2]diazaborinin-3-yl)propanoate (3 mg, 7 µmol, 1.0 equiv.), and DIPEA (7 µL, 42 µmol, 6.0 equiv.) in DMF (0.5 mL) and the reaction stirred at rt for 30 mins. The reaction was concentrated *in vacuo* and purified by prep-HPLC (10-100% MeOH in H_2O (0.01% TFA) to yield the desired product as a purple solid (2 mg, 30 %). ^{1}H NMR (400 MHz, $CDCl_3$) δ 10.38 (s, 1H), 8.99 (s, 1H), 8.02 (s, 1H), 8.00 (d, *J* = 3.0 Hz, 1H), 7.90 (d, *J* = 8.7 Hz, 1H), 7.33 (d, *J* = 2.3 Hz, 1H), 7.24 – 7.19 (m, 2H), 7.16 (d, *J* = 9.8 Hz, 2H), 7.00 (d, *J* = 4.6 Hz, 1H), 6.94 (s, 1H), 6.83 (d, *J* = 4.6 Hz, 1H), 6.79 (d, *J* = 4.0 Hz, 1H), 6.56 (d, *J* = 8.2 Hz, 1H), 6.38 (s, 1H), 6.37 – 6.33 (m, 1H), 6.26 (d, *J* = 4.0 Hz, 1H), 4.49 (s, 2H), 3.58 (s, 4H), 3.54 – 3.48 (m, 10H), 3.43 (q, *J* = 5.2 Hz, 2H), 3.31 (t, *J* = 7.7 Hz, 2H), 2.96 (s, 3H), 2.88 (d, *J* = 0.6 Hz, 3H), 2.60 (t, *J* = 7.7 Hz, 2H). LCMS calculated for $C_{45}H_{50}BF_3N_{11}O_8$ $[M + H]^+$: 940.38. Found: 940.0.

(1*S*,4*R*)-*N*-(15-(5,5-difluoro-7-(1*H*-pyrrol-2-yl)-5*H*-5λ⁴,6λ⁴-dipyrrolo[1,2-*c*:2',1'-*f*][1,3,2]diazaborinin-3-yl)-13-oxo-3,6,9-trioxa-12-azapentadecyl)-4-hydroxy-2,2-dimethyl-4-(5-(3-methyl-5-((4-methylpyrimidin-2-yl)amino)phenyl)thiazol-2-yl)cyclohexane-1-carboxamide (7)

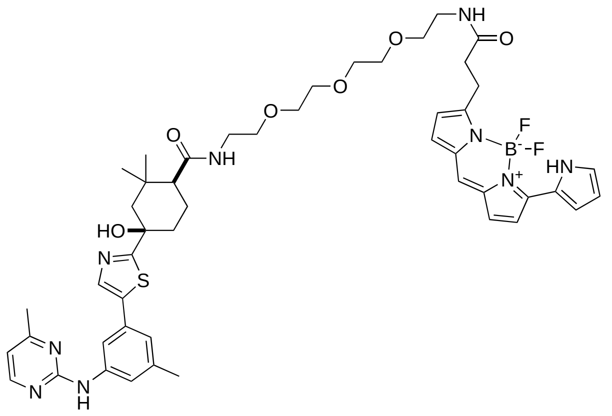

To a flask was added (1*S*,4*R*)-4-hydroxy-2,2-dimethyl-4-(5-(3-methyl-5-((4-methylpyrimidin-2-yl)amino)phenyl)thiazol-2-yl)cyclohexane-1-carboxylic acid (5.0 mg, 11 μmol, 1.0 equiv.), DIPEA (6.2 μL, 35 μmol, 3.2 equiv.), and HATU (5.5 mg, 14 μmol, 1.3 equiv.), in DMF (110 μL, 0.1 M) and the reaction allowed to stir at rt for 5 mins. To this was added *tert*-butyl (2-(2-(2-(2-aminoethoxy)ethoxy)ethoxy)ethyl)carbamate (3.2 mg, 11 μmol, 1.0 equiv.) and the reaction left to stir at rt for 16 hrs to yield the intermediate *tert*-butyl (1-((1*S*,4*R*)-4-hydroxy-2,2-dimethyl-4-(5-(3-methyl-5-((4-methylpyrimidin-2-yl)amino)phenyl)thiazol-2-yl)cyclohexyl)-1-oxo-5,8,11-trioxa-2-azatridecan-13-yl)carbamate. The reaction was concentrated *in vacuo* and deprotected with 20% TFA in CH_2Cl_2 at rt for 1 hr. The intermediate (1*S*,4*R*)-*N*-(2-(2-(2-(2-aminoethoxy)ethoxy)ethoxy)ethyl)-4-hydroxy-2,2-dimethyl-4-(5-(3-methyl-5-((4-methylpyrimidin-2-yl)amino)phenyl)thiazol-2-yl)cyclohexane-1-carboxamide 2,2,2-trifluoroacetate was stirred at rt for 30 mins with 2,5-dioxopyrrolidin-1-yl 3-(5,5-difluoro-7-(1*H*-pyrrol-2-yl)-5*H*-5λ4,6λ4-dipyrrolo[1,2-*c*:2',1'-*f*][1,3,2]diazaborinin-3-yl)propanoate (5.6 mg, 13 μmol, 1.0 equiv.) and DIPEA (14 μL, 35 μmol, 3.2 equiv.) in DMF (130 μL, 0.1 M). The reaction was concentrated *in vacuo* and purified by preparative HPLC (10-100% MeOH in H_2O (+0.01% TFA)) to yield the desired product as a purple solid (6.1 mg, 50% over 3 steps). ^{1}H NMR (400 MHz, MeOD-*d*$_4$) δ 10.67 (s, 1H), 8.24 (d, *J* = 5.4 Hz, 1H), 7.87 (s, 1H), 7.84 – 7.82 (m, 1H), 7.37 – 7.34 (m, 1H), 7.19 – 7.11 (m, 5H), 6.96 (d, *J* = 4.6 Hz, 1H), 6.87 (d, *J* = 4.0 Hz, 1H), 6.77 (d, *J* = 5.4 Hz, 1H), 6.34 – 6.30 (m, 1H), 6.29 (d, *J* = 4.0 Hz, 1H), 3.62 – 3.55 (m, 8H), 3.51 (dt, *J* = 11.2, 5.4 Hz, 4H), 3.37 (t, *J* = 5.5 Hz, 3H), 3.25 (d, *J* = 8.1 Hz, 3H), 2.67 – 2.58 (m, 2H), 2.44 (s, 3H), 2.38 – 2.33 (m, 3H), 2.15 (dd, *J* = 12.6, 2.4 Hz, 1H), 1.97 – 1.89 (m, 3H), 1.71 (d, *J* = 14.3 Hz, 1H), 1.57 – 1.49 (m, 1H), 1.20 (s, 3H), 0.99 (s, 3H). LCMS calculated for $C_{48}H_{59}BF_2N_9O_6S$ [M + H]$^+$: 938.43. Found: 938.1.

(1*S*,4*R*)-*N*-(3-(3-(5,5-difluoro-7-(1*H*-pyrrol-2-yl)-5*H*-5λ^4,6λ^4-dipyrrolo[1,2-*c*:2',1'-*f*][1,3,2]diazaborinin-3-yl)propanamido)propyl)-4-hydroxy-2,2-dimethyl-4-(5-(3-methyl-5-((4-methylpyrimidin-2-yl)amino)phenyl)thiazol-2-yl)cyclohexane-1-carboxamide (8)

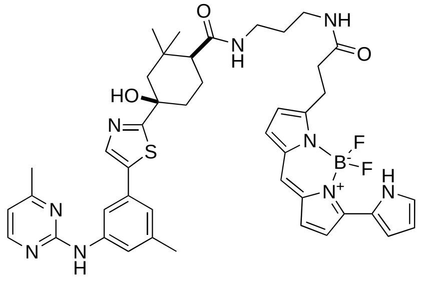

To a flask was added (1*S*,4*R*)-4-hydroxy-2,2-dimethyl-4-(5-(3-methyl-5-((4-methylpyrimidin-2-yl)amino)phenyl)thiazol-2-yl)cyclohexane-1-carboxylic acid (5.0 mg, 11 μmol, 1.0 equiv.), DIPEA (6.2 μL, 35 μmol, 3.2 equiv.), and HATU (5.5 mg, 14 μmol, 1.3 equiv.), in DMF (110 μL, 0.1 M) and the reaction allowed to stir at rt for 5 mins. To this was added *tert*-butyl (3-aminopropyl)carbamate (1.9 mg, 11 μmol, 1.0 equiv.) and the reaction left to stir at rt for 16 hrs to yield the intermediate *tert*-butyl (3-((1*S*,4*R*)-4-hydroxy-2,2-dimethyl-4-(5-(3-methyl-5-((4-methylpyrimidin-2-yl)amino)phenyl)thiazol-2-yl)cyclohexane-1-carboxamido)propyl)carbamate. The reaction was concentrated *in vacuo* and deprotected with 20% TFA in CH_2Cl_2 at rt for 1 hr. The intermediate (1*S*,4*R*)-*N*-(3-aminopropyl)-4-hydroxy-2,2-dimethyl-4-(5-(3-methyl-5-((4-methylpyrimidin-2-yl)amino)phenyl)thiazol-2-yl)cyclohexane-1-carboxamide 2,2,2-trifluoroacetate was stirred at rt for 30 mins with 2,5-dioxopyrrolidin-1-yl 3-(5,5-difluoro-7-(1*H*-pyrrol-2-yl)-5*H*-5λ4,6λ4-dipyrrolo[1,2-*c*:2',1'-*f*][1,3,2]diazaborinin-3-yl)propanoate (5.6 mg, 13 μmol, 1.0 equiv.) and DIPEA (14 μL, 35 μmol, 3.2 equiv.) in DMF (130 μL, 0.1 M). The reaction was concentrated *in vacuo* and purified by preparative HPLC (10-100% MeOH in H_2O (+0.01% TFA)) to yield the desired product as a purple solid (5.9 mg, 65% over 3 steps). ^{1}H NMR (400 MHz, MeOD-*d*$_4$) δ 10.71 (s, 1H), 8.28 (d, *J* = 5.1 Hz, 1H), 7.91 – 7.90 (m, 1H), 7.89 (s, 1H), 7.44 – 7.39 (m, 1H), 7.25 – 7.16 (m, 4H), 7.13 – 7.08 (m, 1H), 7.01 (d, *J* = 4.6 Hz, 1H), 6.92 (d, *J* = 4.0 Hz, 1H), 6.74 (dd, *J* = 5.2, 0.5 Hz, 1H), 6.39 – 6.31 (m, 2H), 3.28 – 3.21 (m, 4H), 3.19 – 3.15 (m, 1H), 2.65 (t, *J* = 7.7 Hz, 2H), 2.44 (s, 3H), 2.37 (s, 3H), 2.33 – 2.20 (m, 1H), 2.16 (dd, *J* = 12.6, 2.6 Hz, 1H), 2.05 – 1.93 (m, 3H), 1.74 – 1.67 (m, 3H), 1.57 (dd, *J* = 13.0, 3.0 Hz, 1H), 1.24 (s, 3H), 1.03 (s, 3H). LCMS calculated for $C_{43}H_{49}BF_2N_9O_3S$ $[M + H]^+$: 820.37. Found: 820.7.

2.5 Purity traces and spectra

***Tert*-butyl 4-(4-((6-bromoimidazo[1,2-*a*]pyrazin-8-yl)amino)phenyl)piperazine-1-carboxylate (9)**

^{1}H NMR

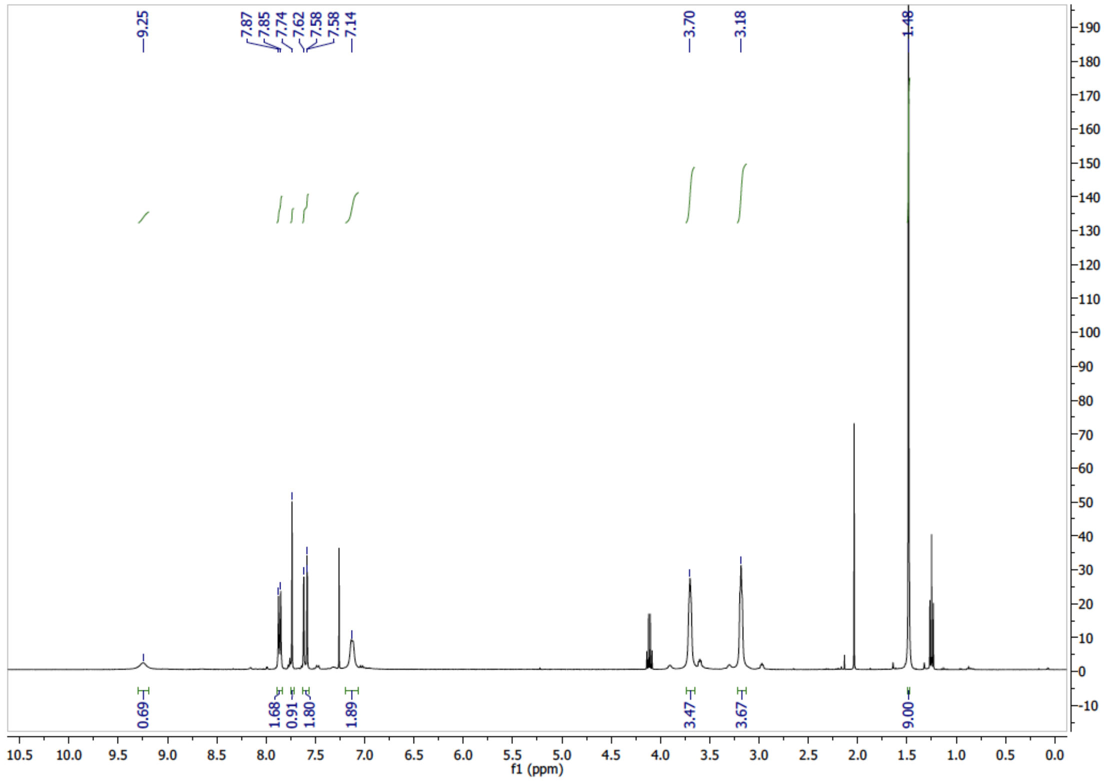

LCMS

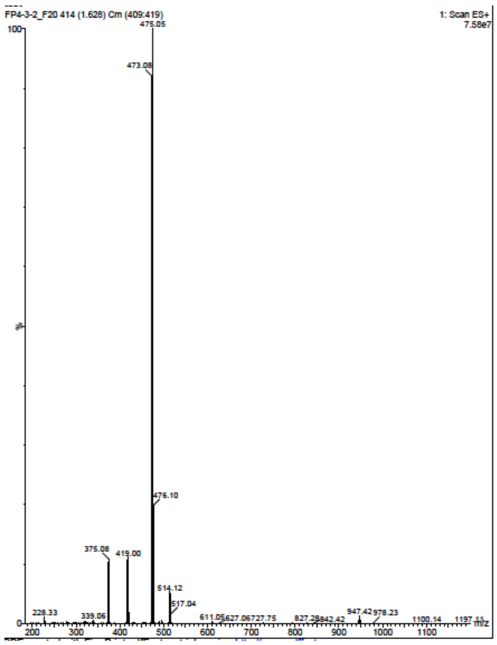

***Tert*-butyl 4-(4-((6-(1*H*-indazol-6-yl)imidazo[1,2-*a*]pyrazin-8-yl)amino)phenyl)piperazine-1-carboxylate (10)**

^{1}H NMR

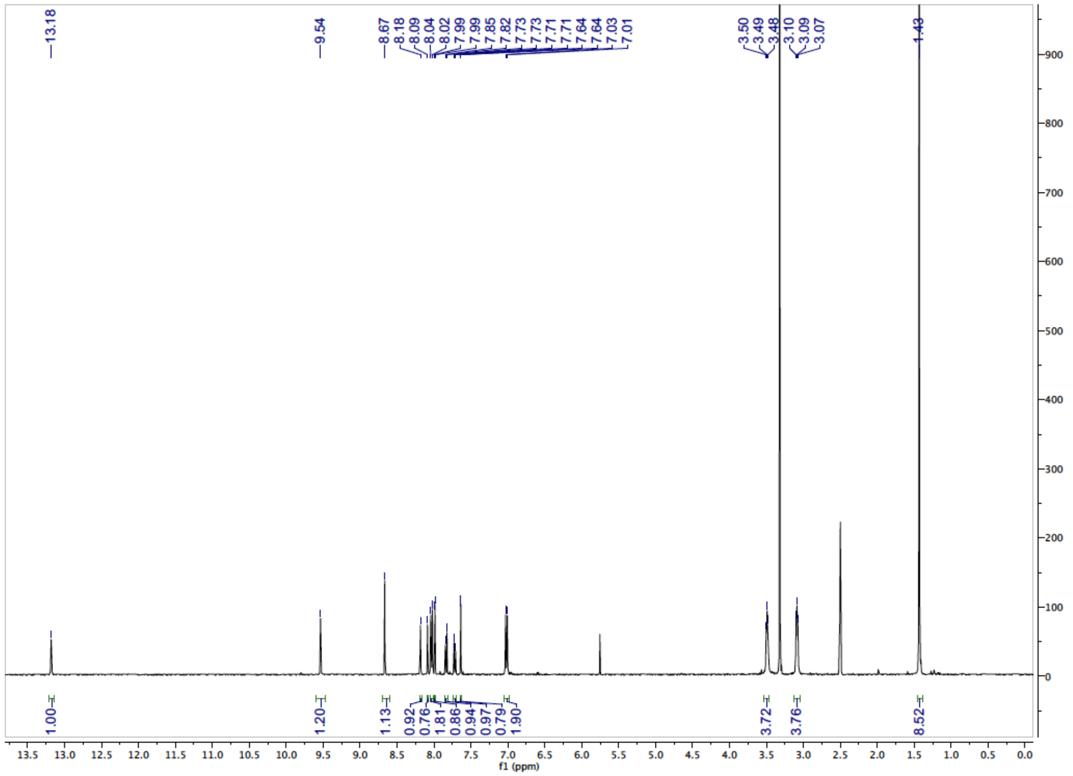

^{13}C NMR

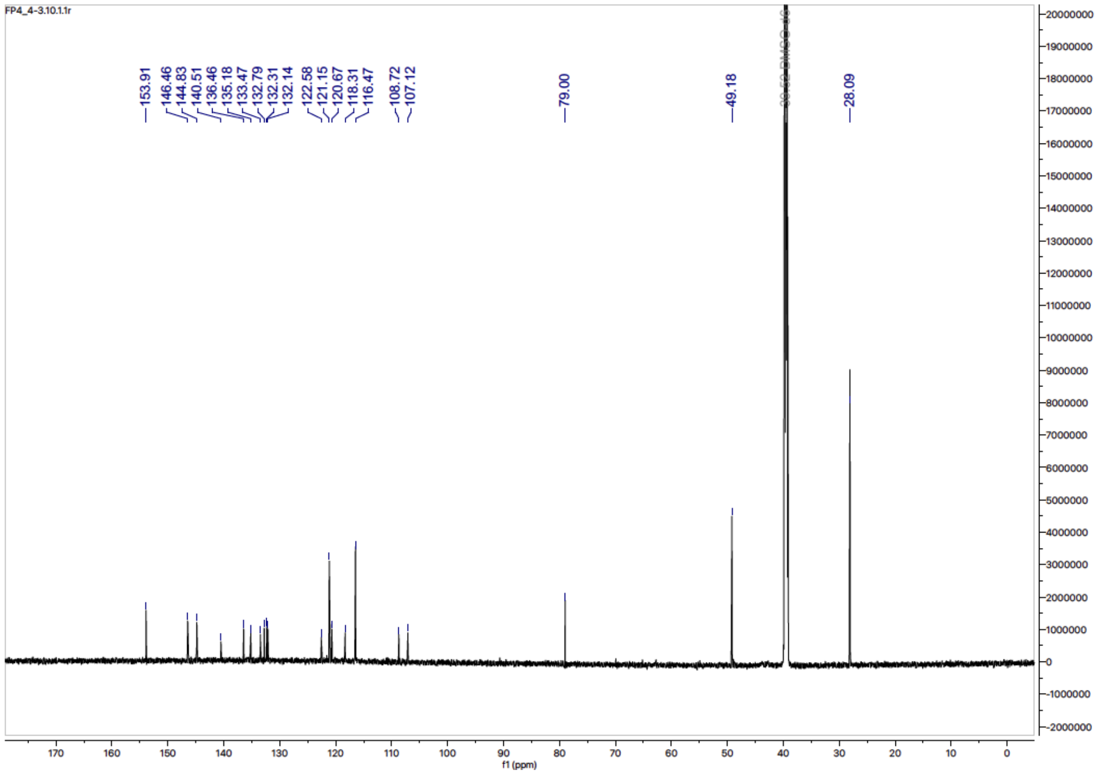

6-(1-methyl-1*H*-indazol-6-yl)-*N*-(4-(piperazin-1-yl)phenyl)imidazo[1,2-*a*]pyrazin-8-amine (3)

^{1}H NMR

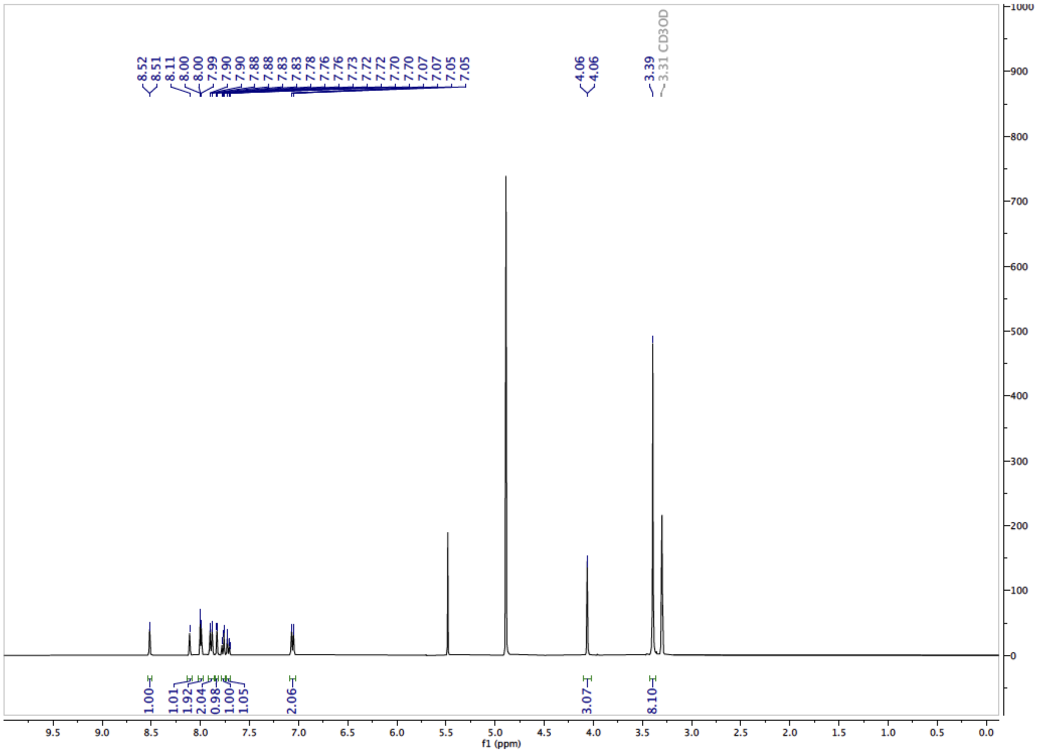

^{13}C NMR

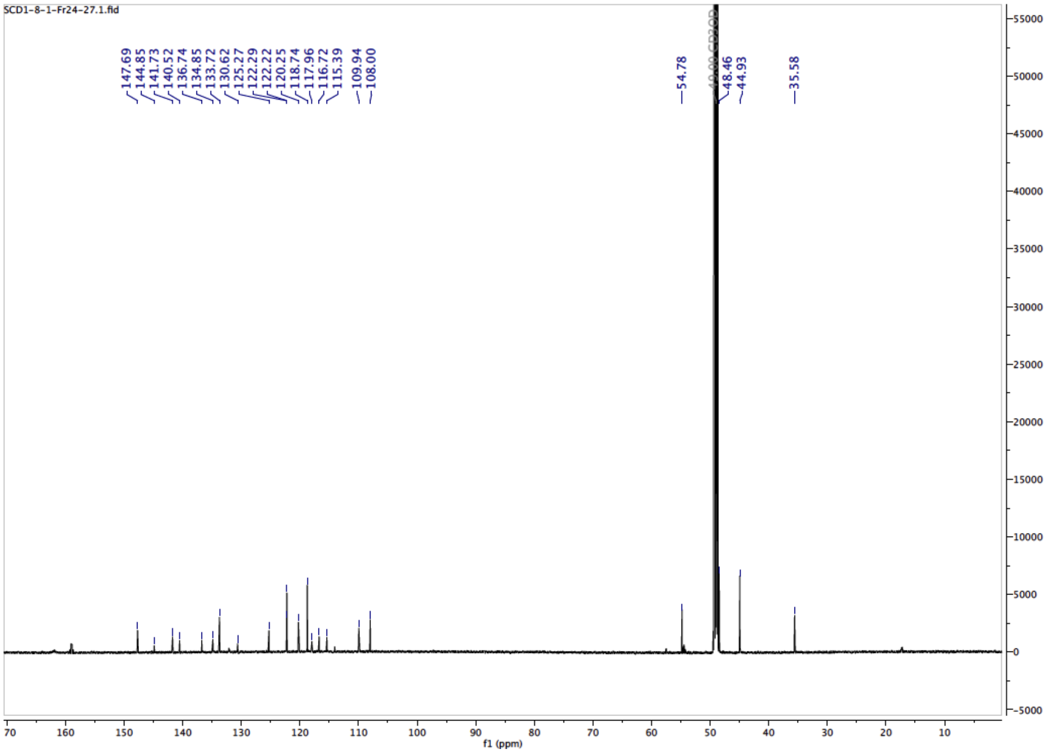

LCMS

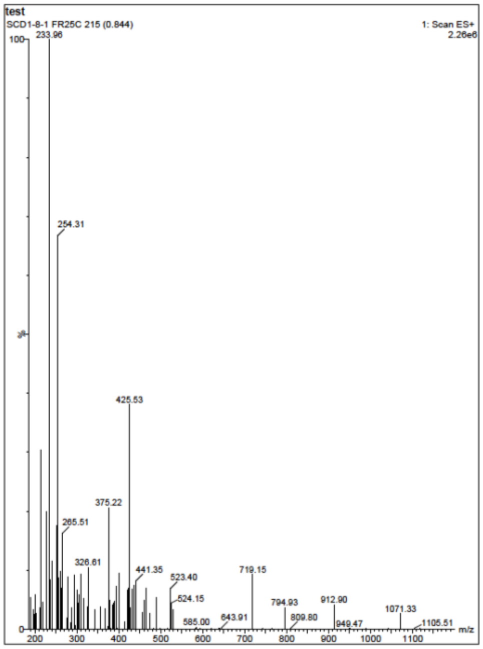

3-(5,5-difluoro-7-(1*H*-pyrrol-2-yl)-5*H*-5λ^4,6λ^4-dipyrrolo[1,2-*c*:2',1'-*f*][1,3,2]diazaborinin-3-yl)-1-(4-(4-((6-(1-methyl-1*H*-indazol-6-yl)imidazo[1,2-*a*]pyrazin-8-yl)amino)phenyl)piperazin-1-yl)propan-1-one (4)

^{1}H NMR

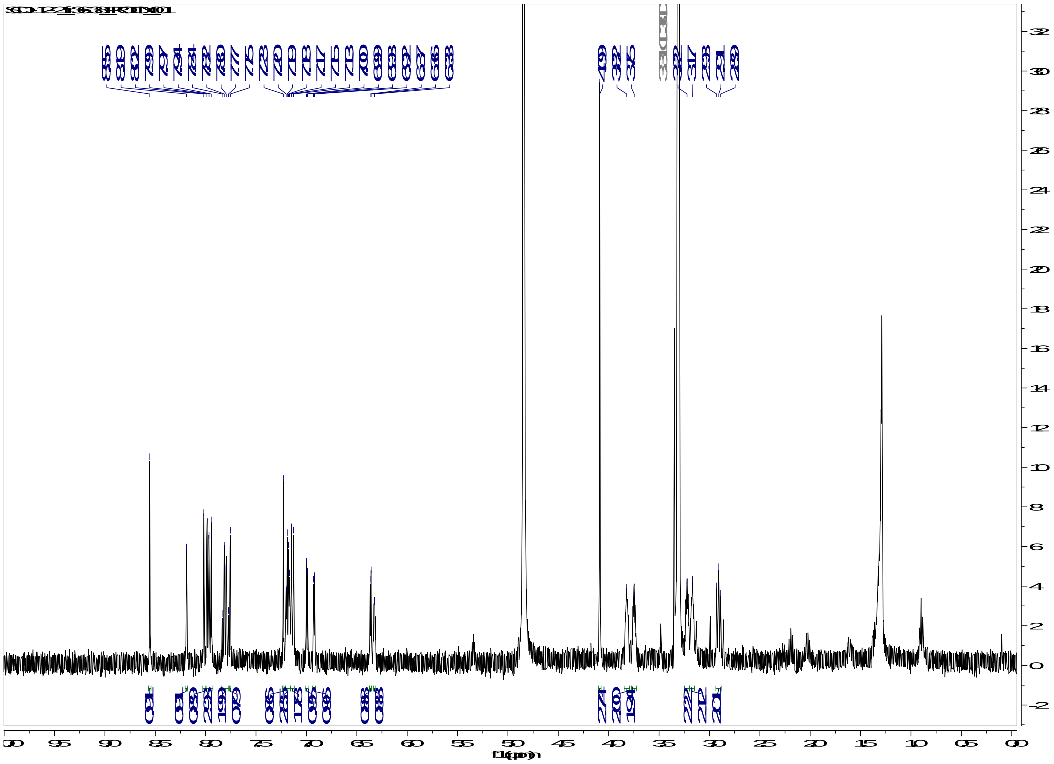

LCMS

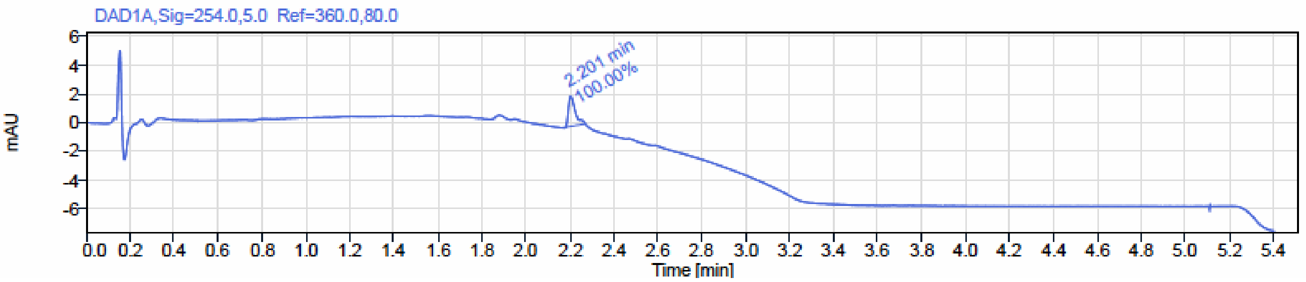
DAD1A,Sig=254.0,5.0 Ref=360.0,80.0
mAU
2.201 min
100.00%
Time [min]

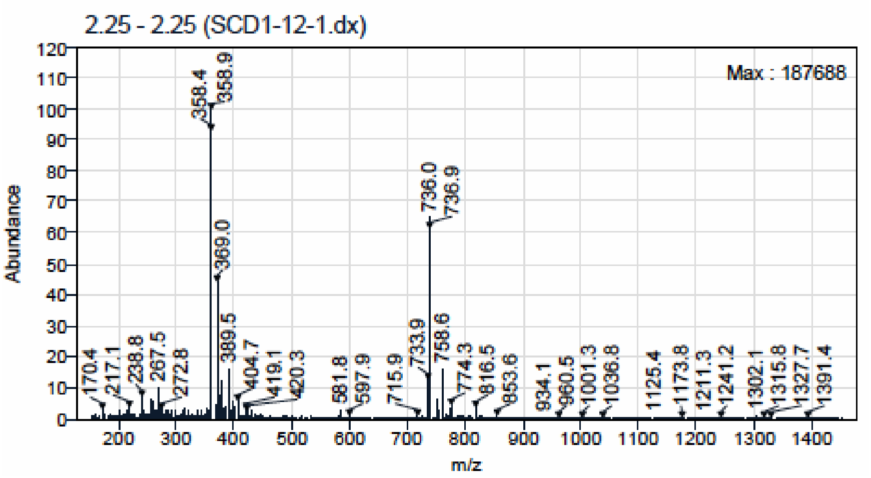
2.25 - 2.25 (SCD1-12-1.dx)
Max : 187688
Abundance
m/z
358.4
358.9
369.0
736.0
736.9
170.4
217.1
238.8
267.5
272.8
389.5
404.7
419.1
420.3
581.8
597.9
715.9
733.9
758.6
774.3
816.5
853.6
934.1
960.5
1001.3
1036.8
1125.4
1173.8
1211.3
1241.2
1302.1
1315.8
1327.7
1391.4

***Tert*-butyl 1-(5,5-difluoro-7-(1*H*-pyrrol-2-yl)-5*H*-5λ^{4},6λ^{4}-dipyrrolo[1,2-*c*:2',1'-*f*][1,3,2]diazaborinin-3-yl)-3-oxo-7,10,13-trioxa-4-azahexadecan-16-oate (11)**

^{1}H NMR

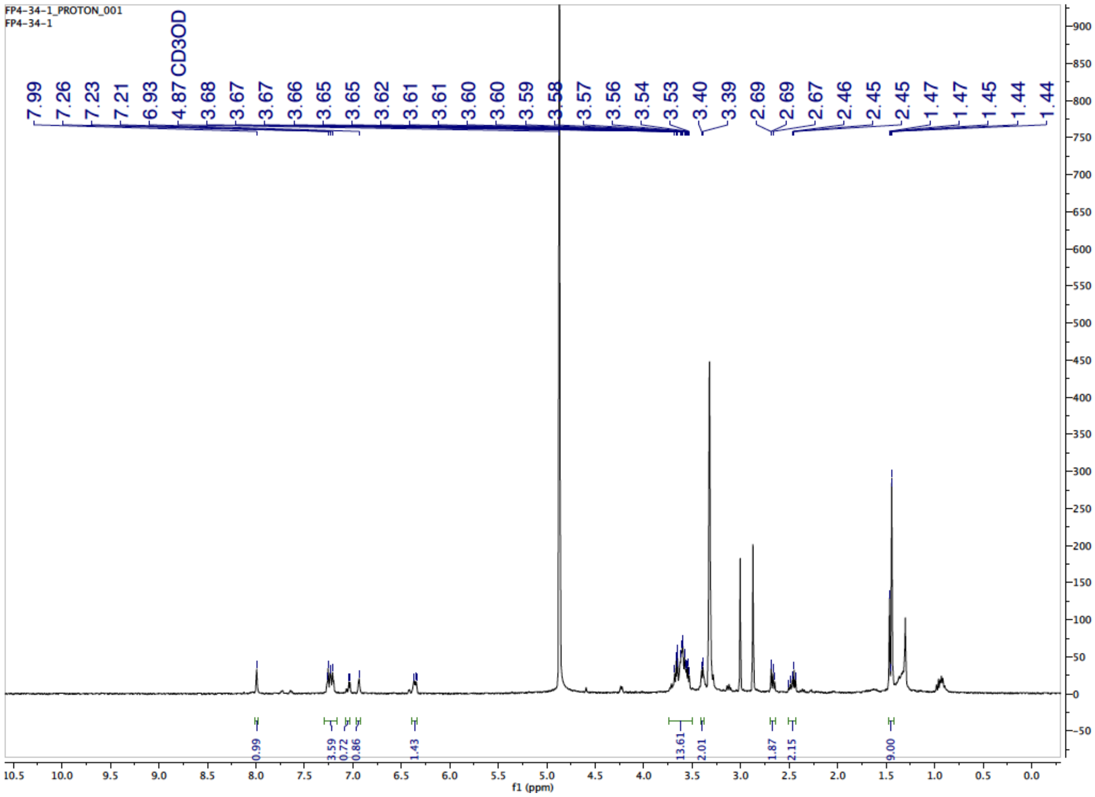

_N_-(2-(2-(2-(3-(4-(4-((6-(1_H_-indazol-6-yl)imidazo[1,2-_a_]pyrazin-8-yl)amino)phenyl)piperazin-1-yl)-3-oxopropoxy)ethoxy)ethoxy)ethyl)-3-(5,5-difluoro-7-(1_H_-pyrrol-2-yl)-5_H_-5λ^4,6λ^4-dipyrrolo[1,2-_c_:2',1'-_f_][1,3,2]diazaborinin-3-yl)propenamide (2)

^{1}H NMR

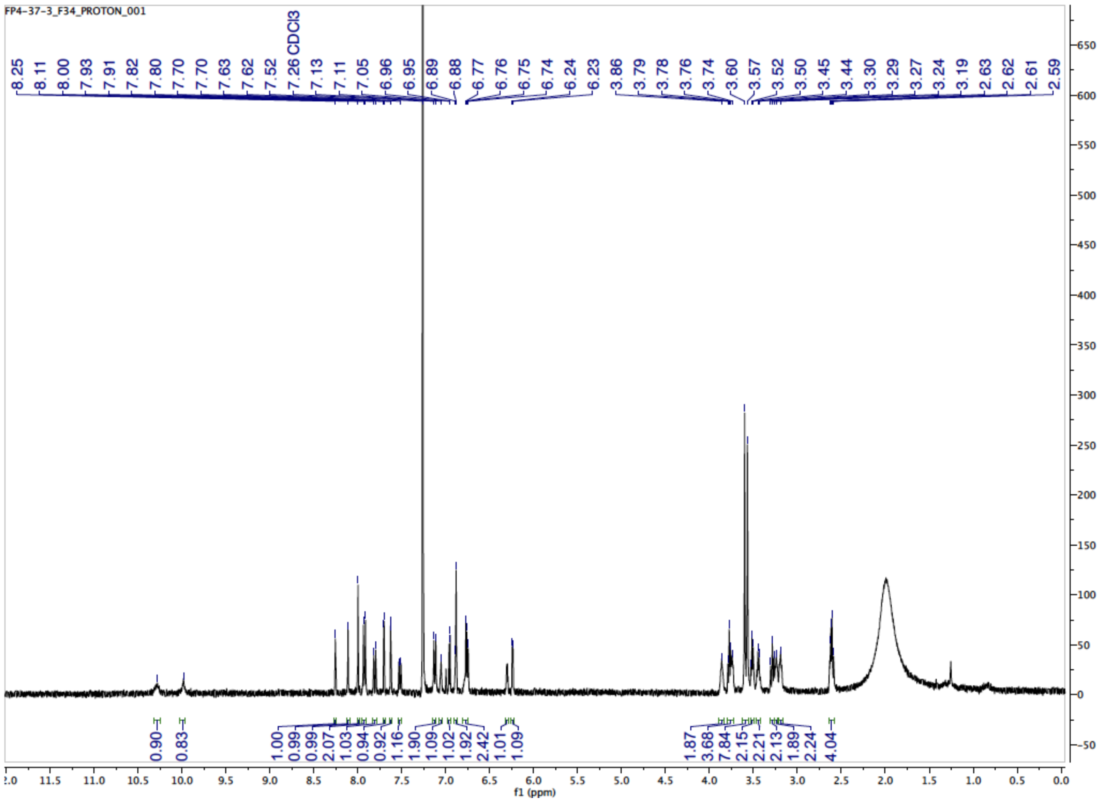

LCMS

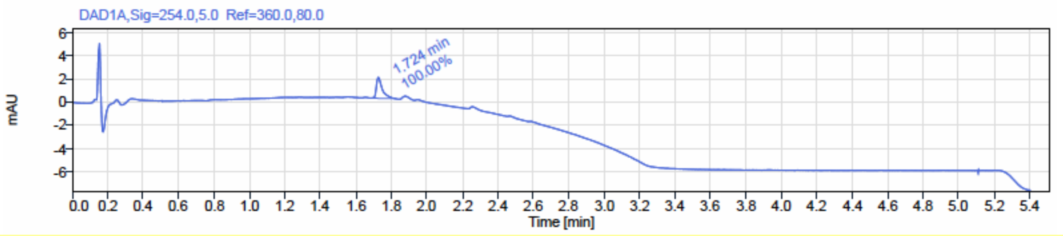

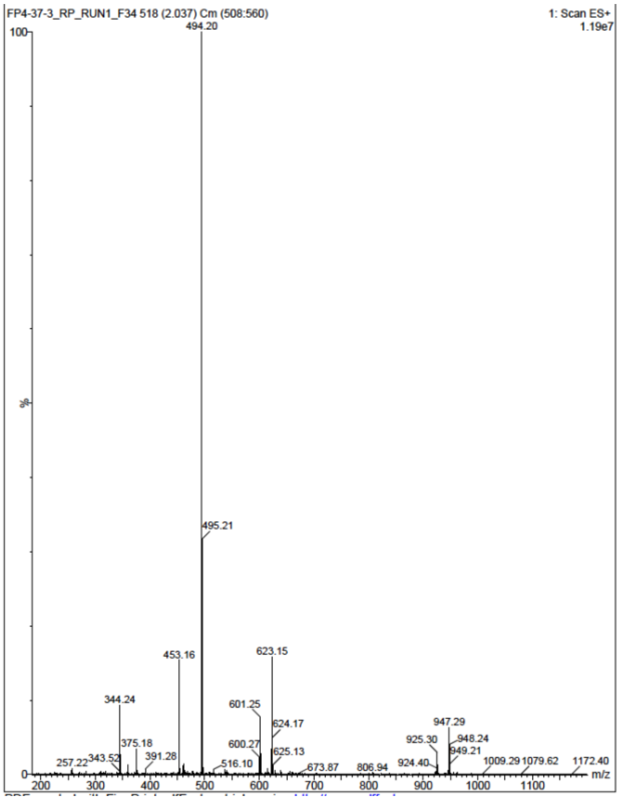

6-((2-chloro-5-fluoropyrimidin-4-yl)amino)-2,2-dimethyl-2*H*-pyrido[3,2-*b*][1,4]oxazin-3(4*H*)-one (13)

^{1}H NMR

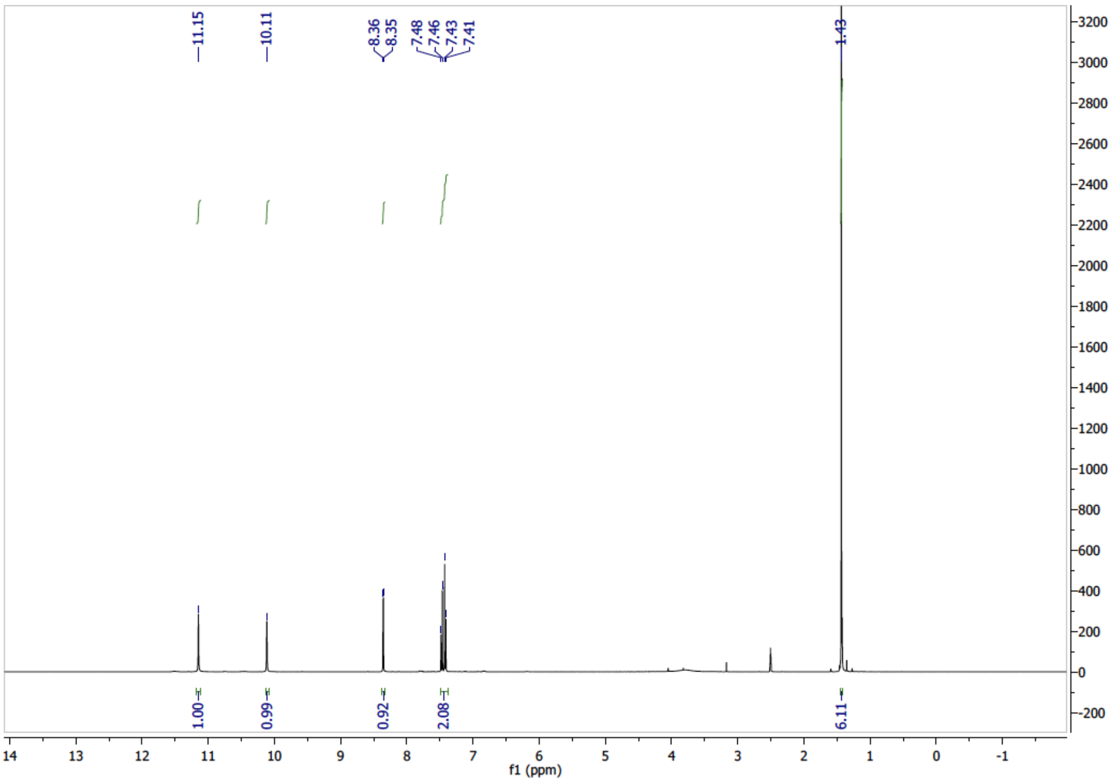

^{13}C NMR

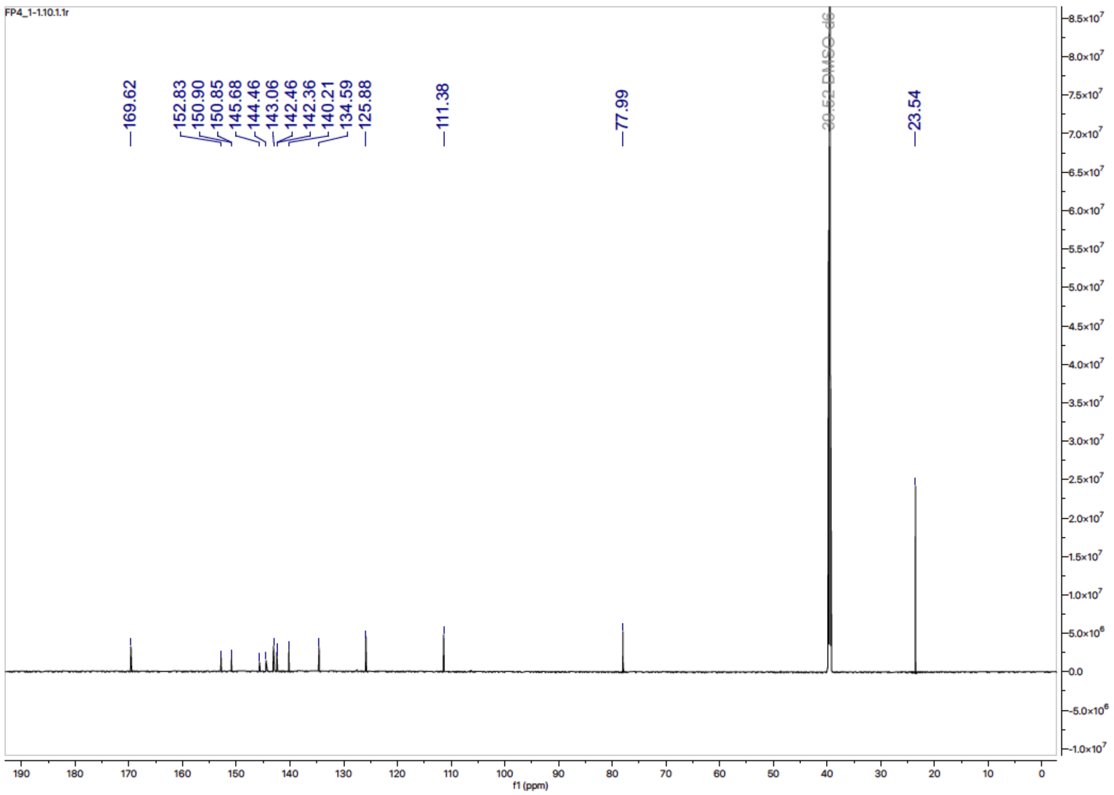
FP4_1-1.10.1.1r
169.62
152.83
150.90
150.85
145.68
144.46
143.06
142.46
142.36
140.21
134.59
125.88
111.38
77.99
39.52 DMSO-d6
23.54
f1 (ppm)

LCMS

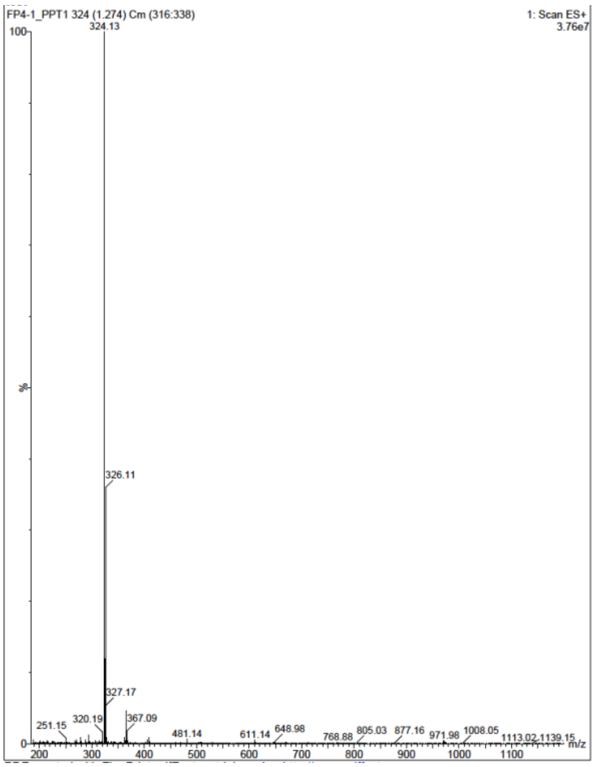
FP4-1_PPT1 324 (1.274) Cm (316:338)
1: Scan ES+
3.76e7
324.13
326.11
327.17
251.15
320.19
367.09
481.14
611.14
648.98
768.88
805.03
877.16
971.98
1008.05
1113.02
1139.15
m/z
%

Ethyl 2-(3-((4-((2,2-dimethyl-3-oxo-3,4-dihydro-2*H*-pyrido[3,2-*b*][1,4]oxazin-6-yl)amino)-5-fluoropyrimidin-2-yl)amino)phenoxy)acetate (14)

^{1}H NMR

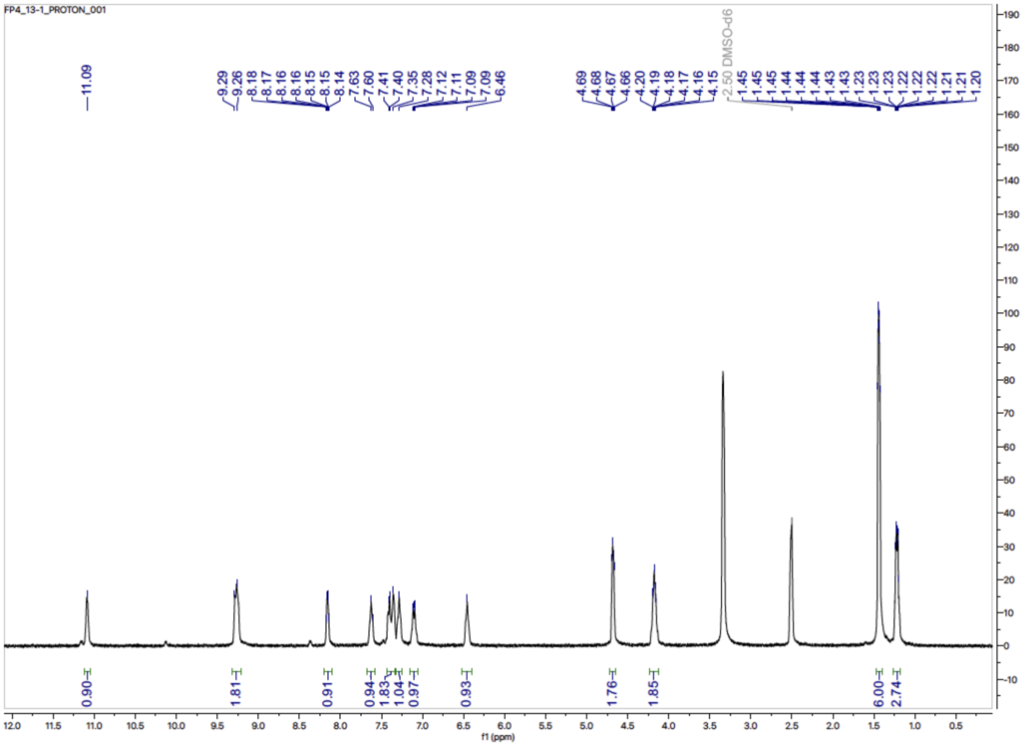

^{13}C NMR

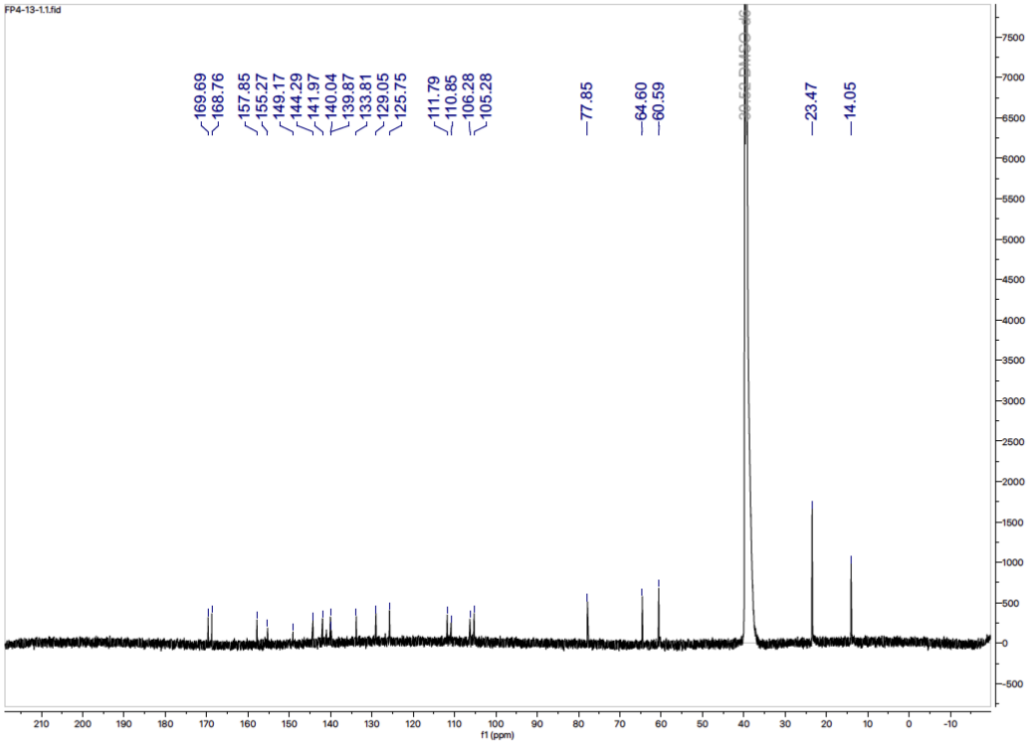

LCMS

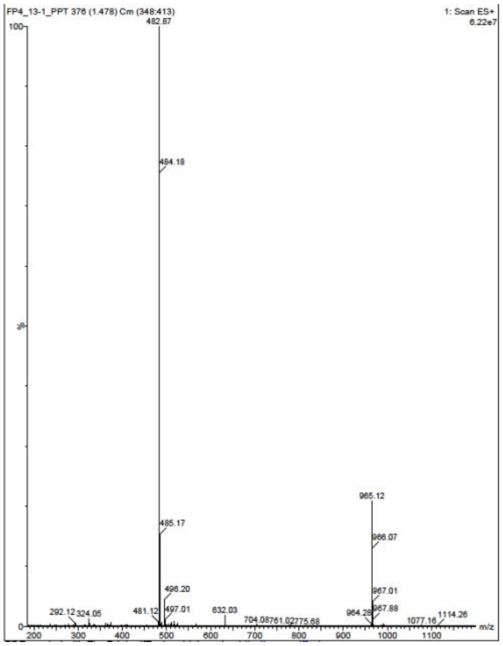

2-(3-((4-((2,2-dimethyl-3-oxo-3,4-dihydro-2*H*-pyrido[3,2-*b*][1,4]oxazin-6-yl)amino)-5-fluoropyrimidin-2-yl)amino)phenoxy)acetic acid (15)

^{1}H NMR

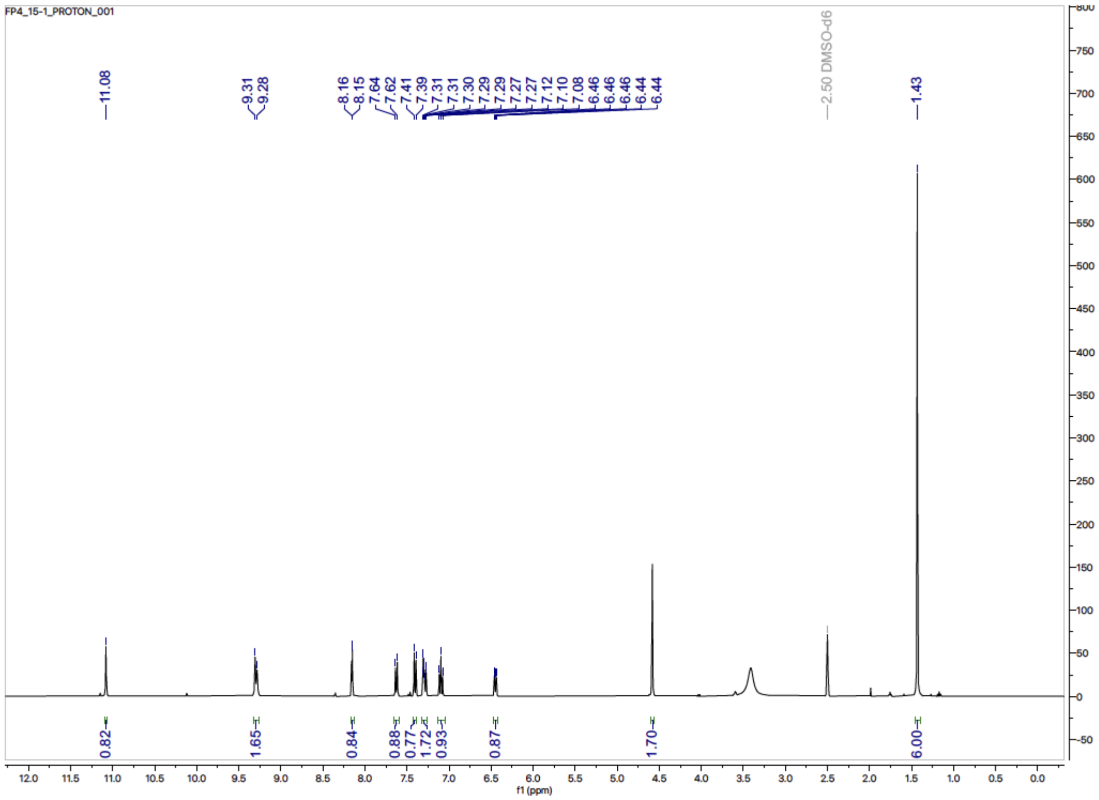

LCMS

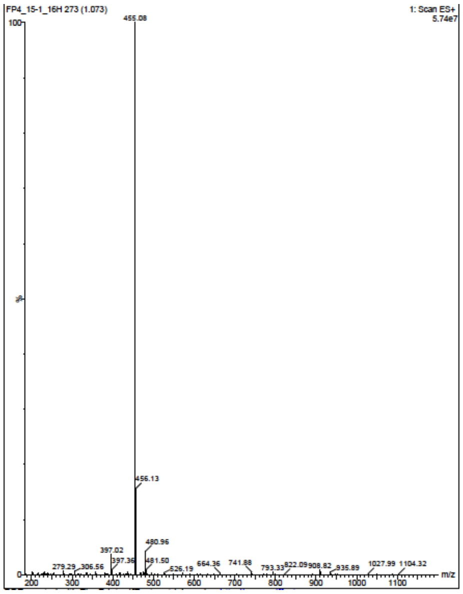

***Tert*-butyl (2-(2-(3-((4-((2,2-dimethyl-3-oxo-3,4-dihydro-2*H*-pyrido[3,2-*b*][1,4]oxazin-6-yl)amino)-5-fluoropyrimidin-2-yl)amino)phenoxy)acetamido)ethyl)carbamate (16)**

^{1}H NMR

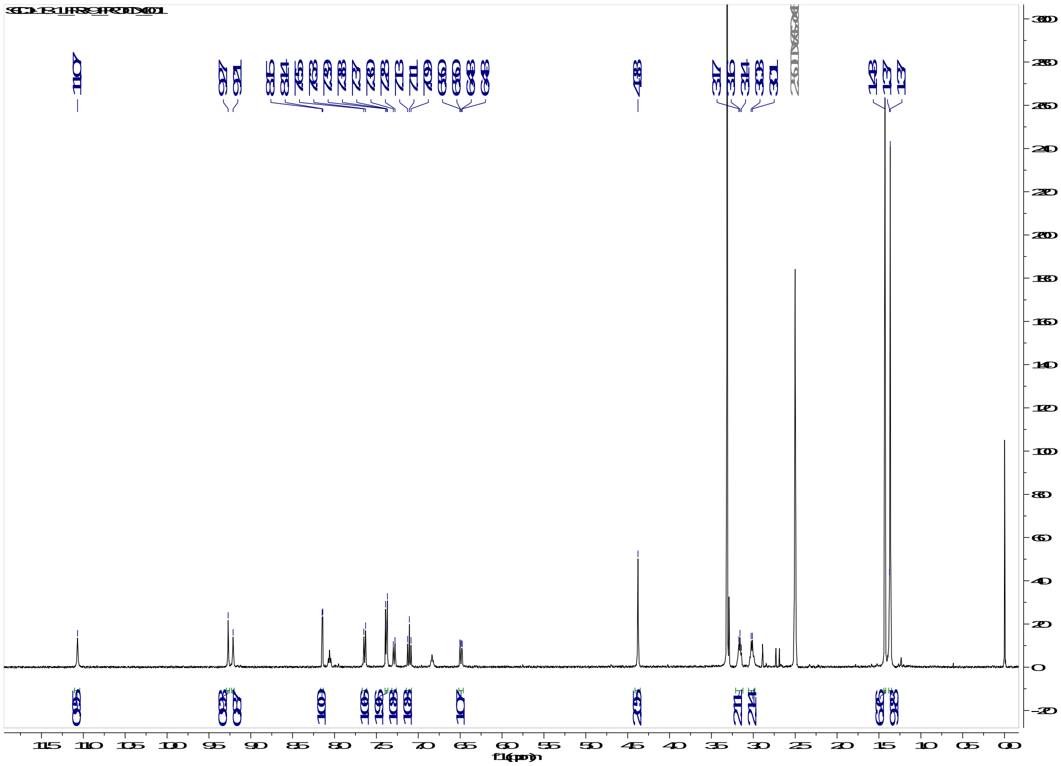

LCMS

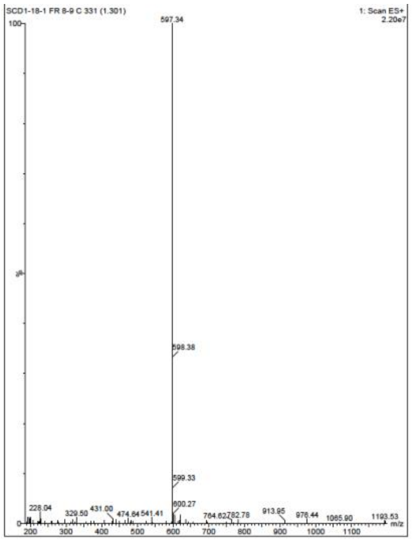

***N*-(2-aminoethyl)-2-(3-((4-((2,2-dimethyl-3-oxo-3,4-dihydro-2*H*-pyrido[3,2-*b*][1,4]oxazin-6-yl)amino)-5-fluoropyrimidin-2-yl)amino)phenoxy)acetamide (17)**

^{1}H NMR

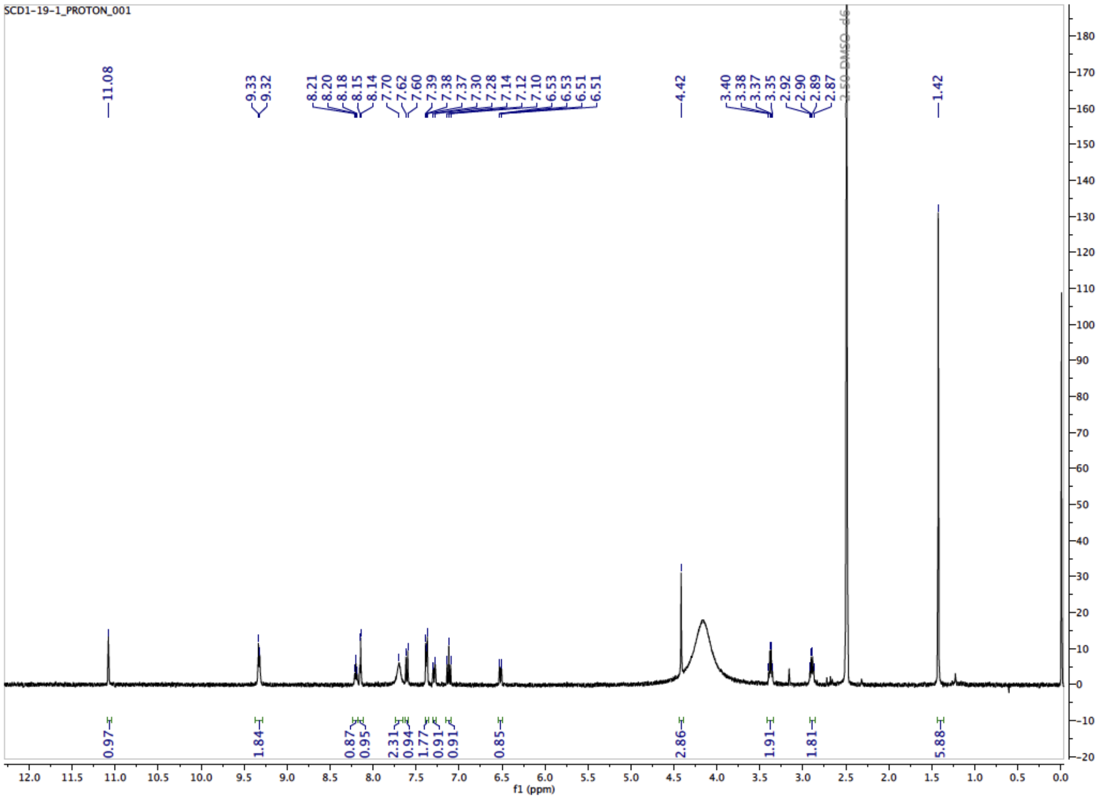

LCMS

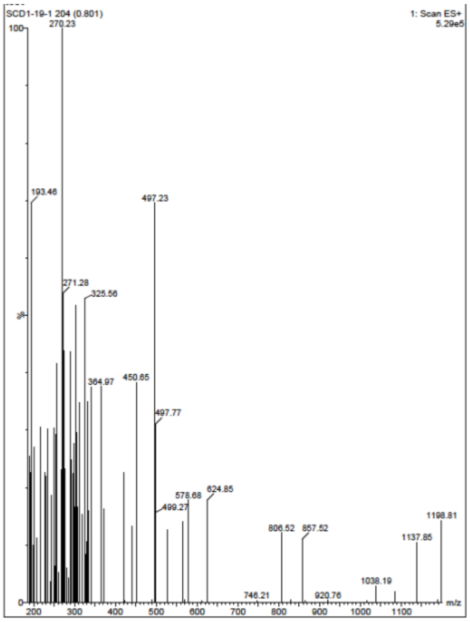

3-(5,5-difluoro-7-(1*H*-pyrrol-2-yl)-5*H*-5λ^4,6λ^4-dipyrrolo[1,2-*c*:2',1'-*f*][1,3,2]diazaborinin-3-yl)-*N*-(2-(2-(3-((4-((2,2-dimethyl-3-oxo-3,4-dihydro-2*H*-pyrido[3,2-*b*][1,4]oxazin-6-yl)amino)-5-fluoropyrimidin-2-yl)amino)phenoxy)acetamido)ethyl)propenamide (6)

^{1}H NMR

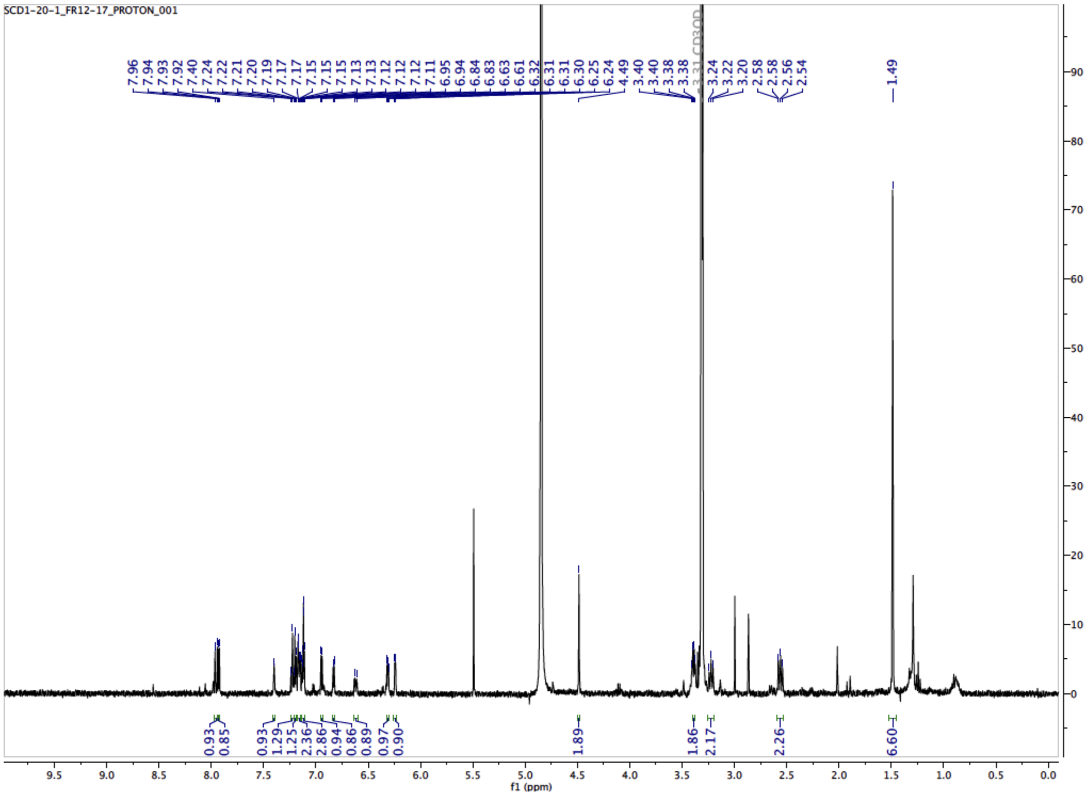

LCMS

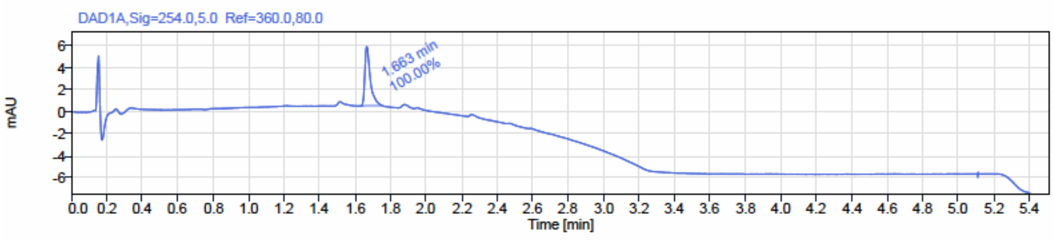

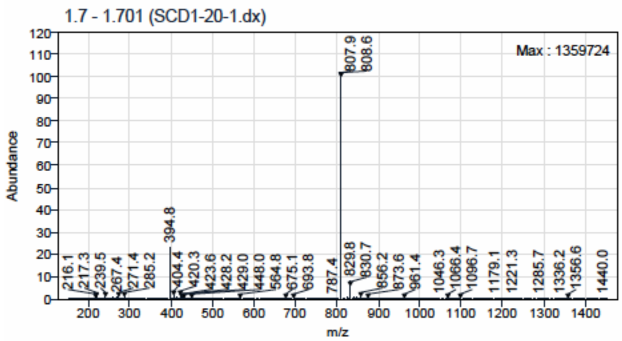

***N*-(2-(2-(2-(2-aminoethoxy)ethoxy)ethoxy)ethyl)-2-(3-((4-((2,2-dimethyl-3-oxo-3,4-dihydro-2*H*-pyrido[3,2-*b*][1,4]oxazin-6-yl)amino)-5-fluoropyrimidin-2-yl)amino)phenoxy)acetamide (18)**

LCMS (Intermediate): *tert*-butyl (1-(3-((4-((2,2-dimethyl-3-oxo-3,4-dihydro-2*H*-pyrido[3,2-*b*][1,4]oxazin-6-yl)amino)-5-fluoropyrimidin-2-yl)amino)phenoxy)-2-oxo-6,9,12-trioxa-3-azatetradecan-14-yl)carbamate)

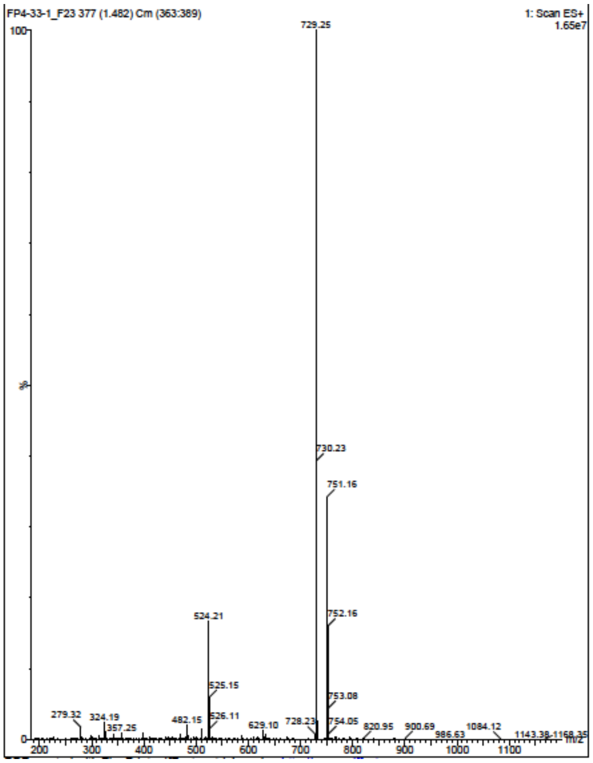

LCMS

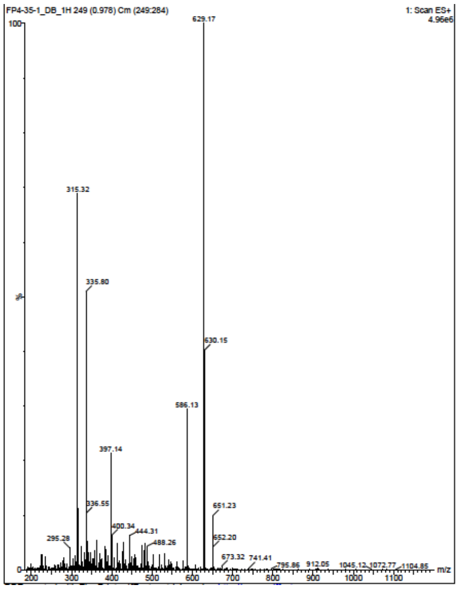

3-(5,5-difluoro-7-(1*H*-pyrrol-2-yl)-5*H*-5λ^4,6λ^4-dipyrrolo[1,2-*c*:2',1'-*f*][1,3,2]diazaborinin-3-yl)-*N*-(1-(3-((4-((2,2-dimethyl-3-oxo-3,4-dihydro-2*H*-pyrido[3,2-*b*][1,4]oxazin-6-yl)amino)-5-fluoropyrimidin-2-yl)amino)phenoxy)-2-oxo-6,9,12-trioxa-3-azatetradecan-14-yl)propenamide (5)

^{1}H NMR

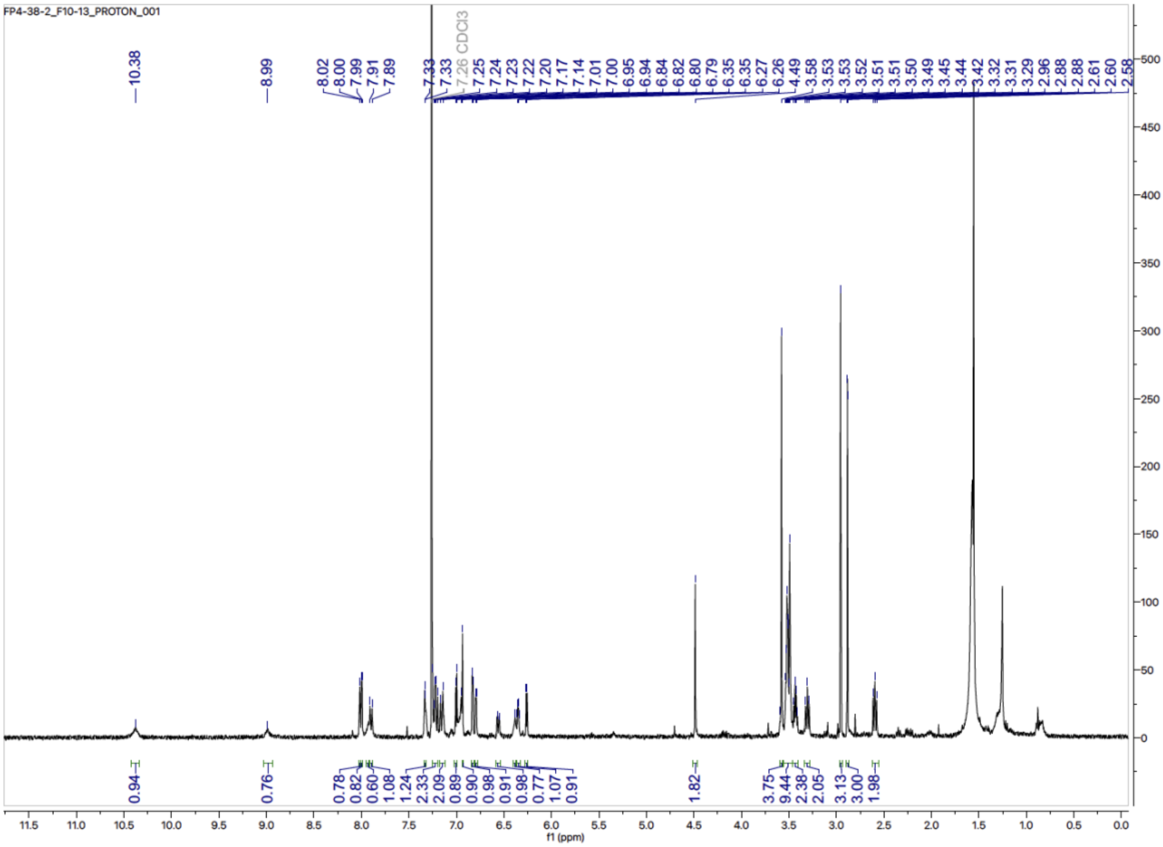

LCMS

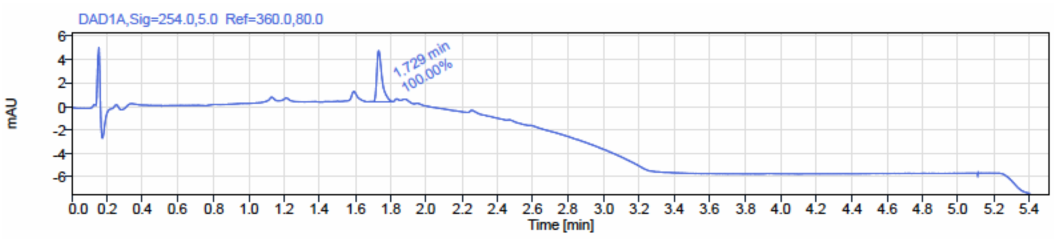

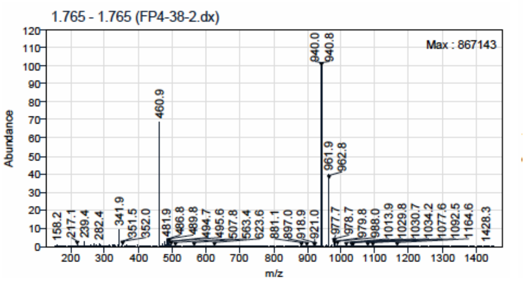

(1*S*,4*R*)-*N*-(15-(5,5-difluoro-7-(1*H*-pyrrol-2-yl)-5*H*-5λ^4,6λ^4-dipyrrolo[1,2-*c*:2',1'-*f*][1,3,2]diazaborinin-3-yl)-13-oxo-3,6,9-trioxa-12-azapentadecyl)-4-hydroxy-2,2-dimethyl-4-(5-(3-methyl-5-((4-methylpyrimidin-2-yl)amino)phenyl)thiazol-2-yl)cyclohexane-1-carboxamide (7)

^{1}H NMR

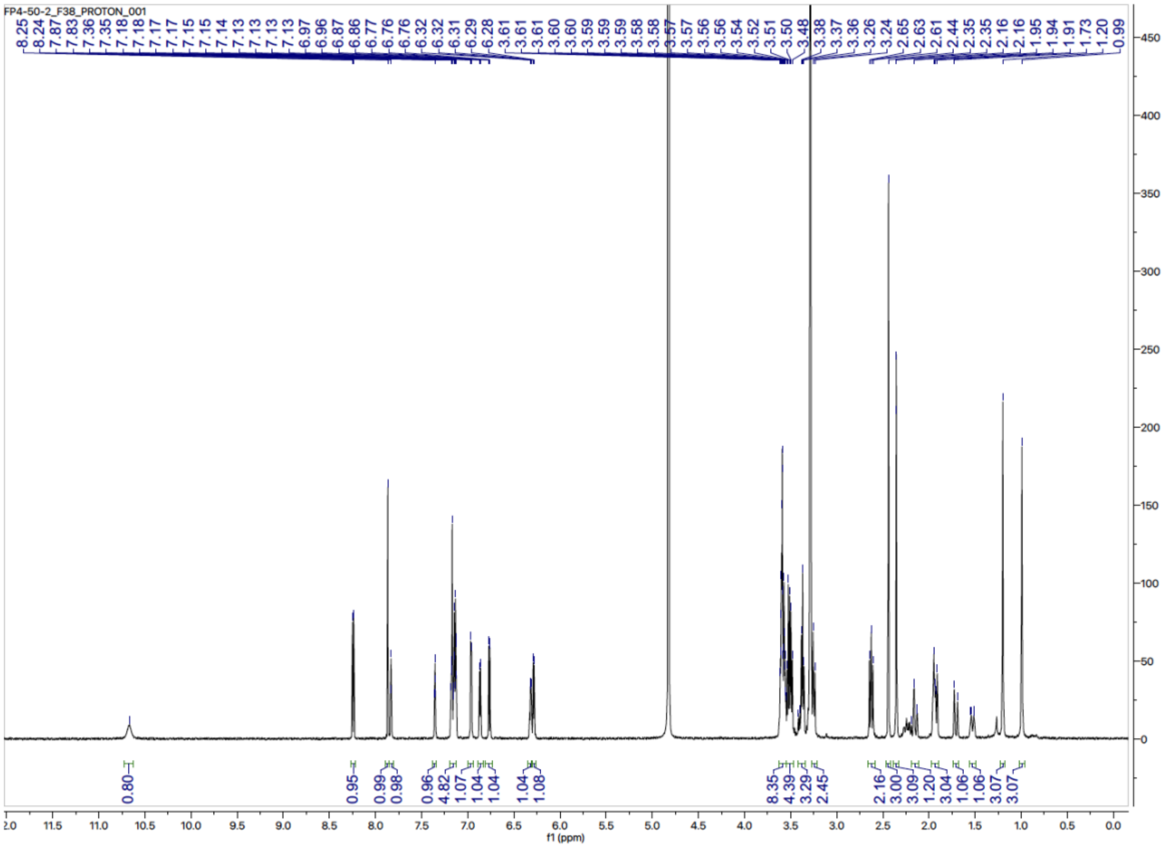

LCMS

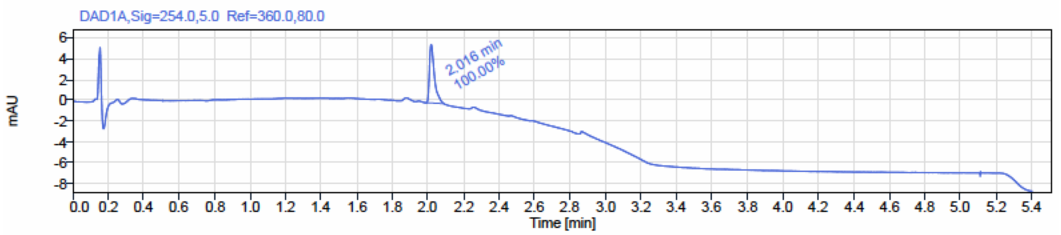

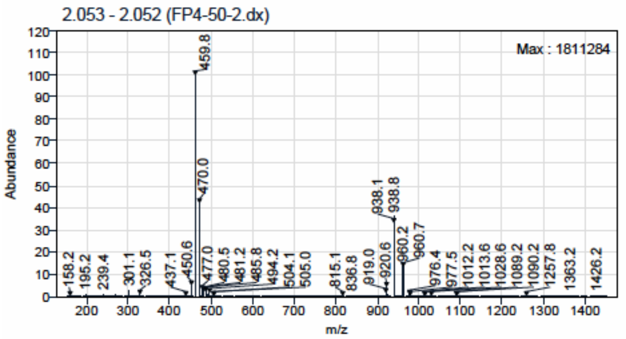

(1*S*,4*R*)-*N*-(3-(3-(5,5-difluoro-7-(1*H*-pyrrol-2-yl)-5*H*-5λ4,6λ4-dipyrrolo[1,2-*c*:2',1'-*f*][1,3,2]diazaborinin-3-yl)propanamido)propyl)-4-hydroxy-2,2-dimethyl-4-(5-(3-methyl-5-((4-methylpyrimidin-2-yl)amino)phenyl)thiazol-2-yl)cyclohexane-1-carboxamide (8)

^{1}H NMR

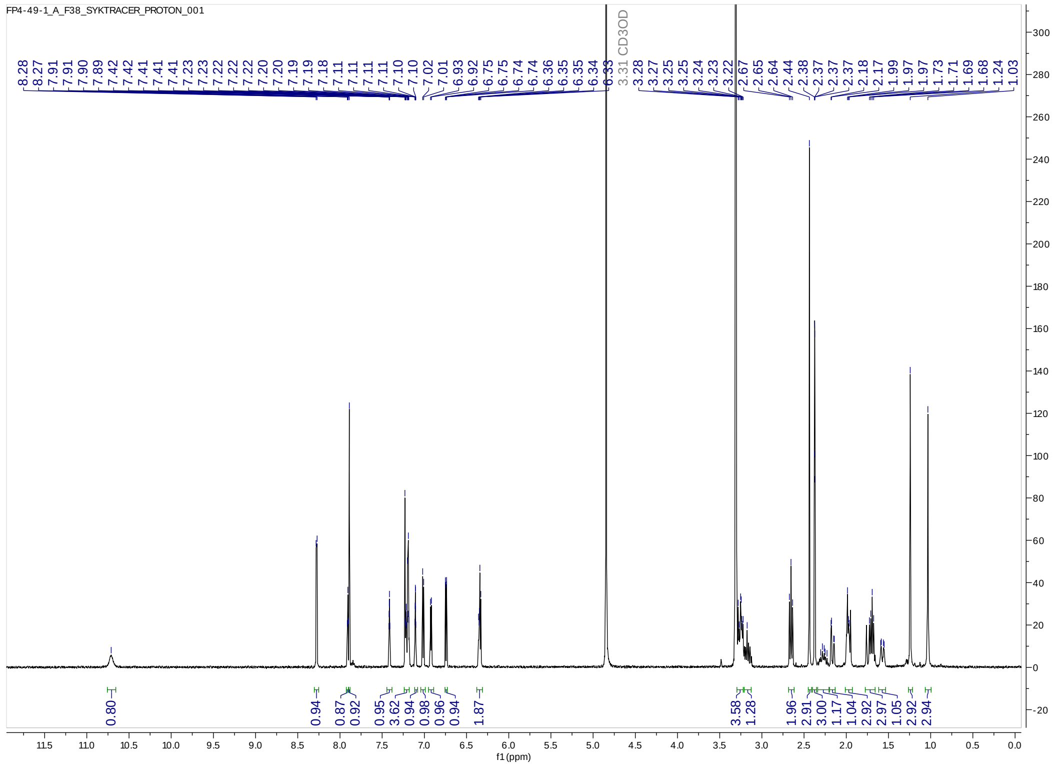

LCMS

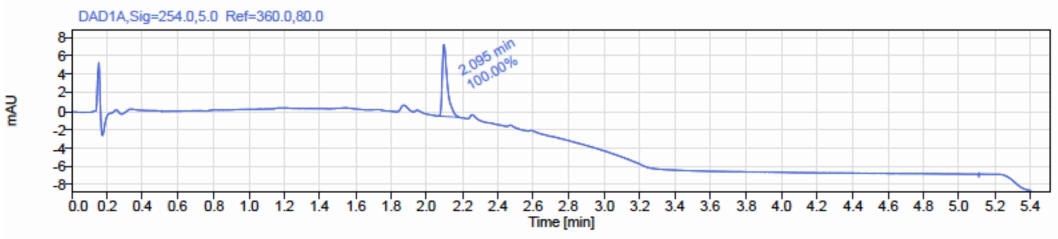
DAD1A,Sig=254.0,5.0 Ref=360.0,80.0
mAU
2.095 min
100.00%
Time [min]

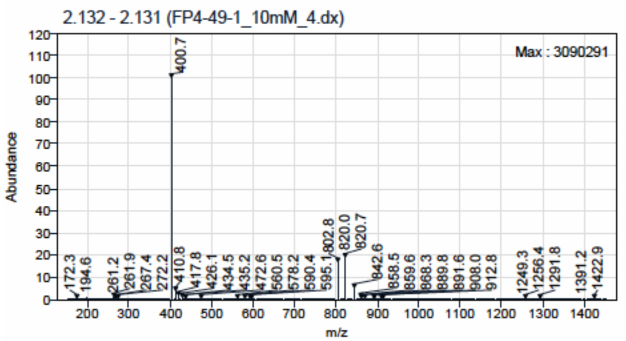
2.132 - 2.131 (FP4-49-1_10mM_4.dx)
Max : 3090291
Abundance
m/z
400.7
802.8
820.0
820.7
